# Genome-Wide Analysis of Microsatellite Markers Based on Sequenced Database in Chinese Spring Wheat (*Triticum aestivum* L.)

**DOI:** 10.1371/journal.pone.0141540

**Published:** 2015-11-04

**Authors:** Bin Han, Changbiao Wang, Zhaohui Tang, Yongkang Ren, Yali Li, Dayong Zhang, Yanhui Dong, Xinghua Zhao

**Affiliations:** 1 College of Bio-engineering, Shanxi University, Taiyuan, China; 2 Biotechnology Research Center, Shanxi Academy of Agricultural Sciences, Taiyuan, China; 3 Institute of Crop Science, Shanxi Academy of Agricultural Sciences, Taiyuan, China; 4 Provincial Key Laboratory of Agrobiology, Institute of Biotechnology, Jiangsu Academy of Agricultural Sciences, Nanjing, China; Sabanci University, TURKEY

## Abstract

Microsatellites or simple sequence repeats (SSRs) are distributed across both prokaryotic and eukaryotic genomes and have been widely used for genetic studies and molecular marker-assisted breeding in crops. Though an ordered draft sequence of hexaploid bread wheat have been announced, the researches about systemic analysis of SSRs for wheat still have not been reported so far. In the present study, we identified 364,347 SSRs from among 10,603,760 sequences of the Chinese spring wheat (CSW) genome, which were present at a density of 36.68 SSR/Mb. In total, we detected 488 types of motifs ranging from di- to hexanucleotides, among which dinucleotide repeats dominated, accounting for approximately 42.52% of the genome. The density of tri- to hexanucleotide repeats was 24.97%, 4.62%, 3.25% and 24.65%, respectively. AG/CT, AAG/CTT, AGAT/ATCT, AAAAG/CTTTT and AAAATT/AATTTT were the most frequent repeats among di- to hexanucleotide repeats. Among the 21 chromosomes of CSW, the density of repeats was highest on chromosome 2D and lowest on chromosome 3A. The proportions of di-, tri-, tetra-, penta- and hexanucleotide repeats on each chromosome, and even on the whole genome, were almost identical. In addition, 295,267 SSR markers were successfully developed from the 21 chromosomes of CSW, which cover the entire genome at a density of 29.73 per Mb. All of the SSR markers were validated by reverse electronic-Polymerase Chain Reaction (re-PCR); 70,564 (23.9%) were found to be monomorphic and 224,703 (76.1%) were found to be polymorphic. A total of 45 monomorphic markers were selected randomly for validation purposes; 24 (53.3%) amplified one locus, 8 (17.8%) amplified multiple identical loci, and 13 (28.9%) did not amplify any fragments from the genomic DNA of CSW. Then a dendrogram was generated based on the 24 monomorphic SSR markers among 20 wheat cultivars and three species of its diploid ancestors showing that monomorphic SSR markers represented a promising source to increase the number of genetic markers available for the wheat genome. The results of this study will be useful for investigating the genetic diversity and evolution among wheat and related species. At the same time, the results will facilitate comparative genomic studies and marker-assisted breeding (MAS) in plants.

## Introduction

Wheat (*Triticum aestivum*) is one of the most important cereals worldwide. The consumption of wheat is greater than that of rice, especially in China and India[1]. Moreover, wheat has long served as a major renewable resource, providing both feed and industrial raw materials [2]. However, after experiencing explosive growth over the past 40 years, the annual increase in wheat yield has begun to slow or even stagnate in most countries of the world[3]. The deterioration of environmental conditions, such as drought, heat and flooding, and the increasing world population have increased the demand for wheat[3]. To face the increasing demand for wheat, it will be important to breed new varieties of wheat that can withstand biotic and abiotic stresses (heat, cold, drought, flooding and so on) while maintaining yields and quality under conservation agriculture management practices [2]. Unlike the traditional process of phenotypic selection, which is too expensive and labor-intensive, new genetic and genomic approaches have been adopted to improve germplasm characterization at the molecular level [4]. During the past decades, great efforts have been made to develop molecular markers in wheat to improvebreeding strategies [5].

Markers can reveal new alleles, as well as original alleles that were reduced in the wheat gene pool during the process of evolution, thereby offering a deeper understanding of wheat during domestication and selection. Due to the very large size and polyploid complexity of wheat genome[6], progress in wheat research has been slow. However, numerous molecular markers including restriction fragment length polymorphisms (RFLPs), random amplified polymorphic DNAs (RAPDs), sequence-tagged sites (STS), DNA amplification fingerprinting (DAF), amplified fragment length polymorphisms (AFLPs), simple sequence repeats (SSRs)/microsatellites, expressed sequence tags (ESTs) and single nucleotide polymorphisms (SNPs) have been used for molecular development, marker-assisted selection and marker validation in various wheat breeding studies [7].

SSRs have become the best choice among markers used in plant breeding programs, as they are practical, convenient, easy to use and inexpensive. Among all available molecular markers, SSRs are easy to score and have wide genomic distribution, codominant inheritance and a multiallelic nature. In addition, SSRs are superior to SNP markers because SSR markers can reveal more information per locus than biallelic SNP markers [8], which explains why SSR markers remain popular. To date, more than 4,000 SSR markers have been developed and used in genetic mapping studies of wheat. These markers enabled the construction of consensus maps or comparative maps by facilitating increasing marker density in specific regions [9]. An amount of SSR markers have been identified from chromosomes in wheat, like 1AL and 5DS[10,11]. Lucas et al have identified 362 SSR markers and 6948 ISBP molecular markers from the long arm of *T*.*aestiivum* chromosome 1A. Then 44 putative markers (eight SSRs, 26 ISBPs and ten ISBPs incorporating SSRs) were tested for polymorphism. 23 (52.3%) were found to be useful. These work will benefit to map chromosomes and further research in wheat marker assisted breeding.

SSRs, which are unevenly distributed in the genomes of prokaryotes and eukaryotes, are tandemly repeated sequences comprising 1–6 base pair (bp) [12]. SSRs derived from expressed sequence tags and genomic libraries are referred to as EST-SSRs and g-SSRs, respectively. To date, numerous SSRs have served as powerful tools to assess genetic diversity, establish core collections, select hybrid parents, study population structures and map or tag functional genes [13]. The polymorphism rate in EST-SSRs is lower than that in g-SSRs [14]. And g-SSRs can serve as valuable complements to EST-SSRs. Numerous EST-SSRs have been generated for wheat, which have revealed high universality between wheat and other cereals, such as barley, maize, rice and sorghum [15,16]. However, few studies have focused on identifying and analyzing g-SSRs in wheat. The availability of the whole draft genome sequence of CSW[6] provides an opportunity to accelerate the process of germplasm evaluation and breeding line identification in wheat breeding programs.

In this study, we identified g-SSRs from the recently sequenced genomic sequence of wheat cv. Chinese spring. The objectives of this study were a) to characterize the density, type and distribution of g-SSR motifs in CSW; b) to develop and analyze Chinese spring genomic SSR markers from a collection of genomic sequences and c) to evaluate the efficiency of these markers in polymorphism identification for application in comparative genomic studies and breeding.

## Materials and Methods

### Plant material

The 23 samples used to validate the polymorphic nature of genic-SSR candidate markers included 20 wheat cultivars and three species of its diploid ancestors (wheat A,B,D -genome progenitor *Triticum urartu*, *Aegilops speltoides Tausch* and *Aegilops tauschii*). The 20 wheat cultivars, *Triticum urartu* and *Aegilops speltoides Tausch* were provided by the Institute of Crop Science, Shanxi Academy of Agricultural Sciences, Taiyuan, China. *Aegilops tauschii* was provided by the Institute of Crop Science, Chinese Academy of Agricultural Sciences, Beijing, China.

### Source of genomic sequences

The genome sequence of model wheat (*Triticum aestivum* cv. Chinese spring) was obtained in FASTA format from URGI (https://urgi.versailles.inra.fr/download/iwgsc/). A total of 10,603,760 sequences were downloaded and studied.

### SSR mining and primer design

The identification and localization of g-SSRs were carried out using MIcroSAtellite (MISA, http://www.pgrc.ipk-gatersleben.de/misa) and Primer 3.0 for large-scale primer design. The criteria used to search SSRs with the MISA script were as follows: motifs between two and six nucleotides long, with a minimum of ten repeats for dinucleotides, seven repeats for trinucleotides, five repeats for tetranucleotides and four repeats for penta- and hexanucleotides. The major parameters for primer design were as follows: primer length, 18–22 bp, with 20 bp being optimal; PCR product size, 100–800 bp; an annealing temperature of 50–65°C, with 57°C being optimal; and a optimal GC content of 50%, with 45% being the minimum.

### Analysis of SSR polymorphism

Analysis of the uniqueness and specificity of the designed SSR markers in the Chinese spring genome was performed using the re-PCR strategy (http://www.ncbi.nlm.nih.gov/tools/epcr/). Re-PCR can be used to map STSs (sequencing tagged site) or short primers in sequence database. It is a version of e-PCR searching for STSs within DNA sequences. Those parameters were: re-PCR—S hash-file—n1 –g1 –r +. Subsequently, the corresponding amplicons were analyzed and the previously obtained SSR markers were classified as definitely polymorphic or monomorphic. Polymorphic SSR markers amplified multiple identical loci in the Chinese spring genome, while monomorphic markers tended to amplify one locus. These data were analyzed with Excel microsoftware and plotted.

### The validation of monomorphic SSR markers in the CSW genome

A total of 45 pairs of g-SSR primers were selected randomly for the validation of the designed monomorphic SSR markers in the CSW genome. Genomic DNA was extracted from fresh, young leaves of CSW using an improved cetyltrimethyl ammonium bromide (CTAB) method[17]. After extraction, the DNA quality and concentration were further assessed using a eppendorf biophotometer. Polymerase chain reaction (PCR) was performed in a total volume of 20.0 μl containing 1 μl of 50 ng/μl template DNA, 2 μl of 10× PCR buffer containing 20 mM MgCl2, 0.4 mM of dNTPs, 0.3 U of Taq polymerase and sterile distilled water and 0.8 μl of 10 μmol/L each of forward and reverse primers. The reactions were performed using the following conditions: 94°C for 2 min; 35 cycles of 94°C for 40 s, 55°C for 45 s, and 72°C for 60 s; and a final step at 72°C for 7 min. Then, 2 μl of the PCR product and a 600bp molecular size marker were loaded onto an 8% denaturing polyacrylamide (PAGE) gel in 1×TBE buffer, run at 100 V, and visualized using silver staining. SSR analysis was performed at least twice to confirm primer amplification.

Phylogenetic relationship[18] among 20 wheat cultivars and three species of its diploid ancestors was constructed in a dendrogram coefficients using the program of NTSYS-pc Version 2.10 to estimate the SSRs monomorphic SSR markers.

## Results

### Identification and characterization of SSRs in the whole genome of CSW

**Fig 1:**
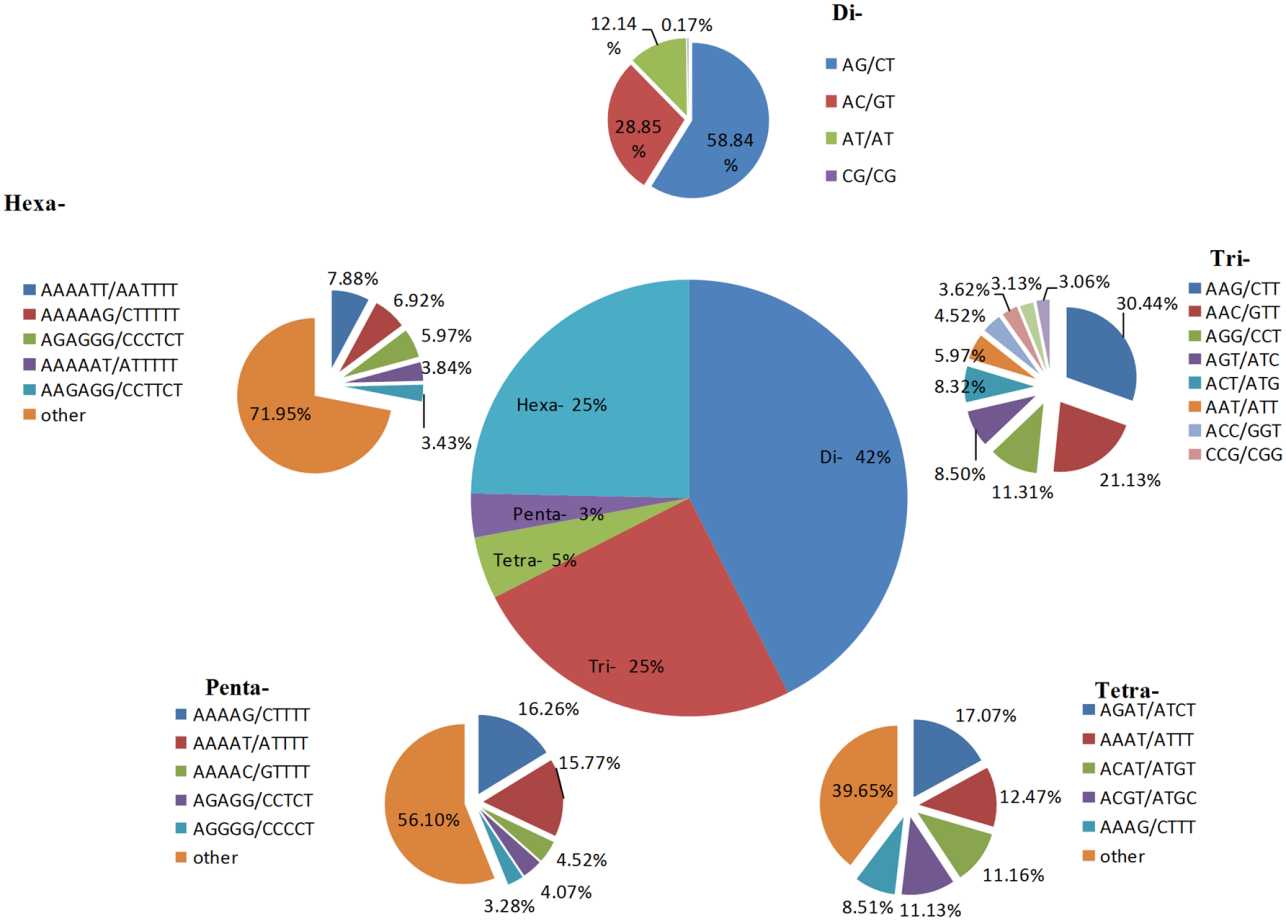
Distribution of repeat types in the Chinese spring wheat.

A total of 364,347 SSRs were identified from among 10,603,760 sequences covering 9,932,960,273 bp of the Chinese spring genome, which were present at a density of 36.68 SSR/Mb. We detected 488 types of SSR motifs in total. Among these types, there were four, 10, 32, 102 and 340 types of di- to hexanucleotide repeats, respectively. The number of each type of motif increased with increasing length, as shown in Table 1. The percentage of dinucleotides was the lowest, while the percentage of hexanucleotides was the highest, accounting for 0.82% and 69.67% of the total, respectively. We further characterized the identified SSRs (Fig 1). Among the different unit sizes, dinucleotide repeats dominated over other types of repeats, accounting for approximately 42.52% of the genome. The density of SSRs decreased stepwise with increasing motif length (di- to pentanucleotide), except for hexanucleotide repeats. The density of tri- to hexanucleotide repeats was 24.97%, 4.62%, 3.25% and 24.65% among all SSRs, respectively. Surprisingly, we found that the ratio of trinucleotides was nearly equal to that of hexanucleotides. Among dinucleotide repeats, AG/CT had the highest occurrence (58.84%), followed by AC/GT (28.85%)and AT/AT (12.14%)). CG/CG (0.17%) were so rare that they could almost be discounted. Among trinucleotide repeats, AAG/CTT made up the highest proportion (30.44%), followed by nine types of repeats, including AAC/GTT (21.13%), AGG/CCT (11.31%), AGT/ATC (8.50%), ACT/ATG (8.32%), AAT/ATT (5.97%), ACC/GGT (4.52%), CCG/CGG (3.62%), AGC/CGT (3.13%) and ACG/CTG (3.06%). The most common tetranucleotide repeats were AGAT/ATCT (17.07%), AAAT/ATTT (12.47%) and ACAT/ATGT (11.16%). AT-rich repeat patterns were the most abundant among penta- and hexanucleotides, such as AAAAG/CTTTT, AAAAT/ATTTT and AAAAC/GTTTT for pentanucleotides and AAAATT/AATTTT, AAAAAG/CTTTTT and AGAGGG/CCCTCT for hexanucleotides (S1 Table).

**Table 1:** Characteristics of SSRs in CSW.

SSR mining		Total	
Total length of analyzed equences(bp)		9932960273	
Number of identified SSRs		364347	
SSR/Mb		36.68	
**Type**	**Kinds**	**number**	**percentage(%)**
Di	4	154913	42.52
Tri	10	90960	24.97
Tetra	32	16823	4.62
Penta	102	11828	3.25
Hexa	340	89823	24.65
Total	488	364347	100.00

### Characterization of SSRs on each chromosome of CSW

**Fig 2:**
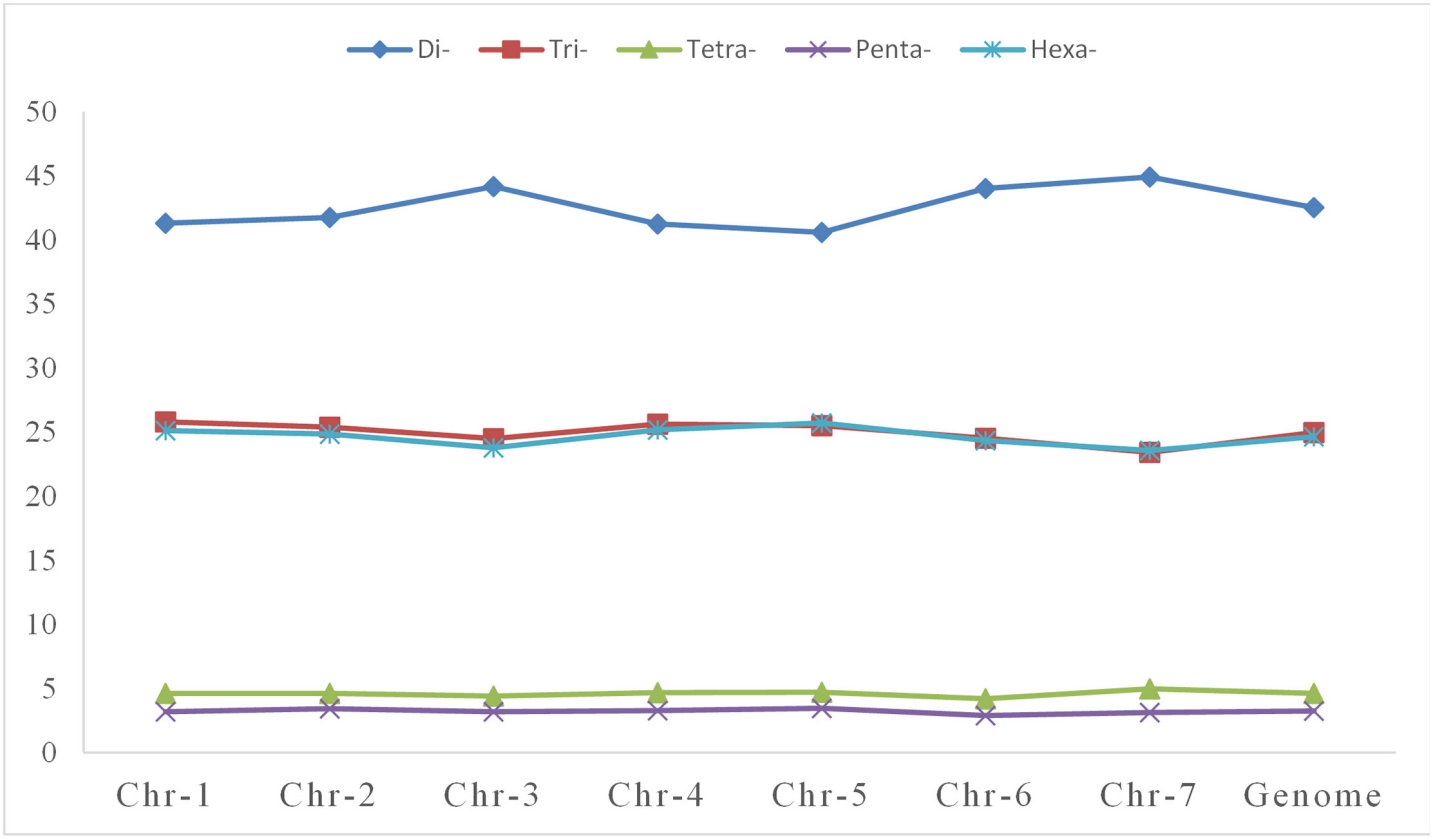
Frequency (%) of di- to hexanucleotide motifs in the Chinese spring wheat.

We then analyzed the distribution of SSRs on each chromosome of the Chinese spring genome (Table 2). The number of SSRs on each chromosome was as follows: 1A to 7A: 14,703, 20,822, 14,289, 20,728, 17,477, 16,211 and 17,699, respectively; 1B to 7B: 18,876, 25,495, 24,717, 20,049, 21,523, 18,005 and 18,814, respectively; 1D to 7D: 12,264, 17,406, 11,670, 11,052, 14,072, 12,302 and 16,173, respectively. Therefore, chromosome 2B and 3B had the largest number of SSRs among Chinese spring chromosomes, followed by chromosome 5B, 2A and 4A, while chromosome 4D contained the fewest SSRs. The top three longest chromosomes are2B, 3B and 4A and the shortest is 4D, which is in accordance with the number of SSRs per chromosome. Overall, the differences in the densities of SSRs on different chromosomes were not significant, ranging from 41.28 SSR/Mb to 32.06 SSR/Mb. The density on chromosome 2D was the highest (41.28 SSR/Mb), while that on chromosome 3A was the lowest (32.06 SSR/Mb). As mentioned above, the average density of SSRs on the entire genome was 36.68 SSR/Mb, which is close to the density on chromosomes 4B and 5B. We then analyzed the repeats on each chromosome (Table 3). The percentage of di-, tri-, tetra-, penta- and hexanucleotide repeats on every chromosome (and even on the whole genome) was nearly identical. The percentage of dinucleotide repeats was the highest, followed by tri-, hexa- and tetranucleotide repeats, while the percentage of pentanucleotide repeats was so low that they could almost be discounted. The ratios of tri- and hexanucleotides were nearly equivalent (Fig 2). In addition, AG/CT and AC/GT were the most abundant dinucleotide repeats on each chromosome, while AAG/CTT, AAC/GTT, AGG/CCT, AGT/ATC and ACT/ATG were the most abundant trinucleotide repeats on each chromosome. The largest proportion of tetranucleotide repeats included AGAT/ATCT, AAAT/ATTT, ACAT/ATGT and ACGT/ATGC, and the most abundant penta- and hexanucleotide repeats were AAAAN and AAAAAN, respectively (S2 Table).

**Table 2:** Chromosome-wide distribution of SSRs in the CSW genome.

Genome	Chromosome	bp	SSR	SSR/Mb
A	1A	424409652	14703	34.64
	2A	576846926	20822	36.10
	3A	445759266	14289	32.06
	4A	600765939	20728	34.50
	5A	511490084	17477	34.17
	6A	428557670	16211	37.83
	7A	446879367	17699	39.61
B	1B	507010493	18876	37.23
	2B	686985855	25495	37.11
	3B	632916551	24717	39.05
	4B	548472594	20049	36.55
	5B	582228367	21523	36.97
	6B	464691465	18005	38.75
	7B	461943202	18814	40.73
D	1D	379056246	12264	32.35
	2D	421689632	17406	41.28
	3D	327157283	11670	35.67
	4D	325658742	11052	33.94
	5D	380292678	14072	37.00
	6D	351893008	12302	34.96
	7D	428255253	16173	37.76

**Table 3:** Number of different repeat classes in Chinese spring.

SSR repeat	Genome							
	1	2	3	4	5	6	7	Total
Di-	18923	26587	22370	21373	21541	20468	23651	154913
Tri-	11822	16168	12405	13278	13542	11404	12341	90960
Tetra-	2121	2948	2236	2427	2505	1964	2622	16823
Penta-	1464	2187	1621	1708	1837	1353	1658	11828
Hexa-	11513	15833	12044	13043	13647	11329	12414	89823
Total-	45843	63723	50676	51829	53072	46518	52686	364347

### Genome-wide SSR markers development and polymorphism analysis

**Fig 3:**
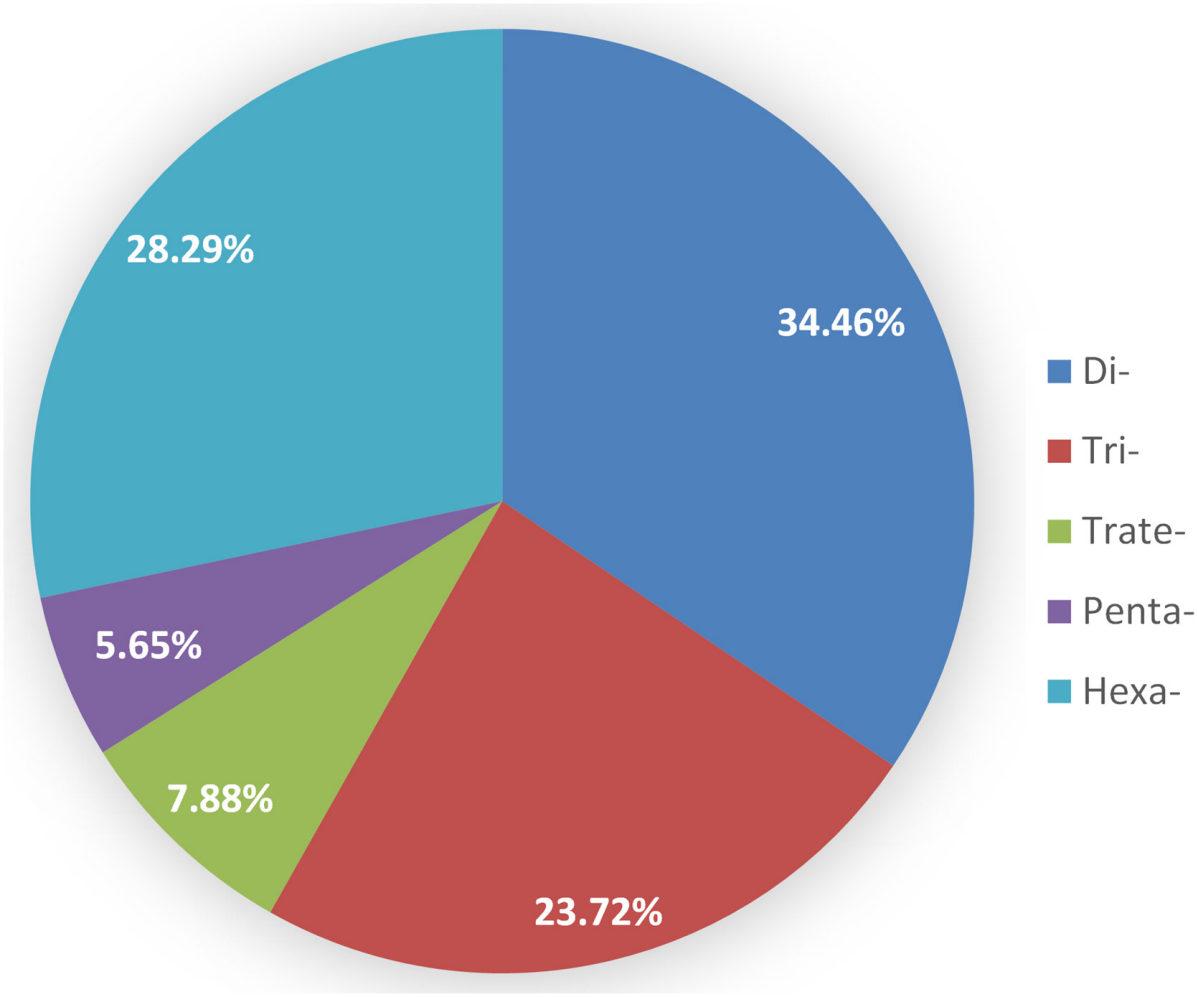
Distribution of monomorphic markers in the Chinese spring wheat genome.

All SSRs were selected for SSR marker development, and a total of 295,267 SSR markers were successfully designed from the 21 chromosomes of CSW, covering the whole genome at a density of 29.73 per Mb. The densities of SSR markers on each chromosome were similar, ranging from 25.58 to 31.34 per Mb (Table 4). Among the chromosomes that contained SSR markers, the highest density of SSR markers was found on chromosome 5D, followed by 2D and 7D. Furthermore, all SSR markers were validated and subjected to polymorphism analysis via re-PCR. Markers that amplified prominent PCR products were classified as either polymorphic or monomorphic based on the number of corresponding loci. Of the markers amplified, 70,564 were monomorphic and 224,703 were polymorphic. The monomorphic markers included 2,387 (3.38%) present in compound formation and 8,177 (99.96%) present in perfect formation, whose dinucleotide motifs (34.46%) were the most common, followed by hexanucleotide (28.29%), trinucleotide (23.72%), tetranucleotide (7.88%) and pentanucleotide motifs (5.65%), respectively (Fig 3). Moreover, we also examined the distribution of monomorphic markers on the Chinese spring chromosomes. Chromosome 3B had 5,387 monomorphic markers, which was considerably higher than that of the other chromosomes, followed by chromosome 2B and 5B, containing 5,112 and 4,346 monomorphic markers, respectively. Chromosome 1D contained the fewest monomorphic markers (2,082), while 3B contained the largest number of di-, tetra-, penta- and hexanucleotide motifs and 2B contained the largest number of trinucleotide motifs. Chromosome 1D had the fewest di- and hexanucleotide motifs, 3D had the fewest tri- and pentanucleotide motifs and 4D had the fewest tetranucleotide motifs (Table 5).

**Table 4:** Summary of SSR markers in the Chinese spring genome.

Genome	Chromosome	bp	SSR marker	SSR marker/Mb
A	1A	424409652	12364	29.13
	2A	576846926	17163	29.75
	3A	445759266	11898	26.69
	4A	600765939	17113	28.49
	5A	511490084	14274	27.91
	6A	428557670	13308	31.05
	7A	446879367	13922	31.15
B	1B	507010493	15589	30.75
	2B	686985855	21001	30.57
	3B	632916551	19583	30.94
	4B	548472594	16250	29.63
	5B	582228367	17570	30.18
	6B	464691465	14126	30.40
	7B	461943202	14343	31.05
D	1D	379056246	10218	26.96
	2D	421689632	13193	31.29
	3D	327157283	8370	25.58
	4D	325658742	9093	27.92
	5D	380292678	11919	31.34
	6D	351893008	10611	30.15
	7D	428255253	13359	31.19
Whole genome		9932960273	295267	29.73

**Table 5:** Characteristics of monomorphic SSR markers in the CSW genome.

Genome	Chromosome	compound	Di-	Tri-	Trate-	Penta-	Hexa-	Total
A	1A	87	883	676	232	155	856	2889
	2A	129	1306	964	340	213	1171	4123
	3A	93	977	626	225	146	787	2854
	4A	122	1319	890	319	225	1113	3988
	5A	78	932	619	258	180	776	2843
	6A	102	1153	791	236	161	960	3403
	7A	140	1257	696	322	170	916	3501
B	1B	131	1162	810	232	165	864	3364
	2B	182	1629	1308	359	278	1356	5112
	3B	266	1812	1242	377	305	1385	5387
	4B	115	1112	816	233	189	961	3426
	5B	133	1352	1113	329	259	1160	4346
	6B	141	1234	674	199	155	813	3216
	7B	147	1278	727	261	179	888	3480
D	1D	51	629	480	172	152	598	2082
	2D	110	1230	784	240	206	991	3561
	3D	71	749	478	192	97	613	2200
	4D	62	704	514	156	117	620	2173
	5D	80	948	711	244	180	853	3016
	6D	41	703	532	166	135	681	2258
	7D	106	1128	719	283	183	923	3342
Total		2387	23497	16170	5375	3850	19285	70564

### The validation of monomorphic SSR markers in the CSW genome

**Fig 4:**
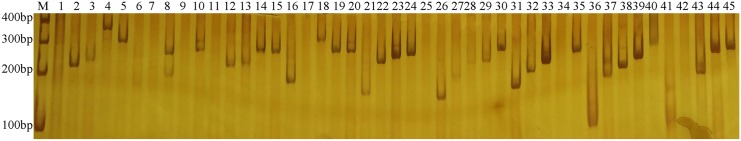
The validation of monomorphic SSR markers in the CSW genome.

**Fig 5:**
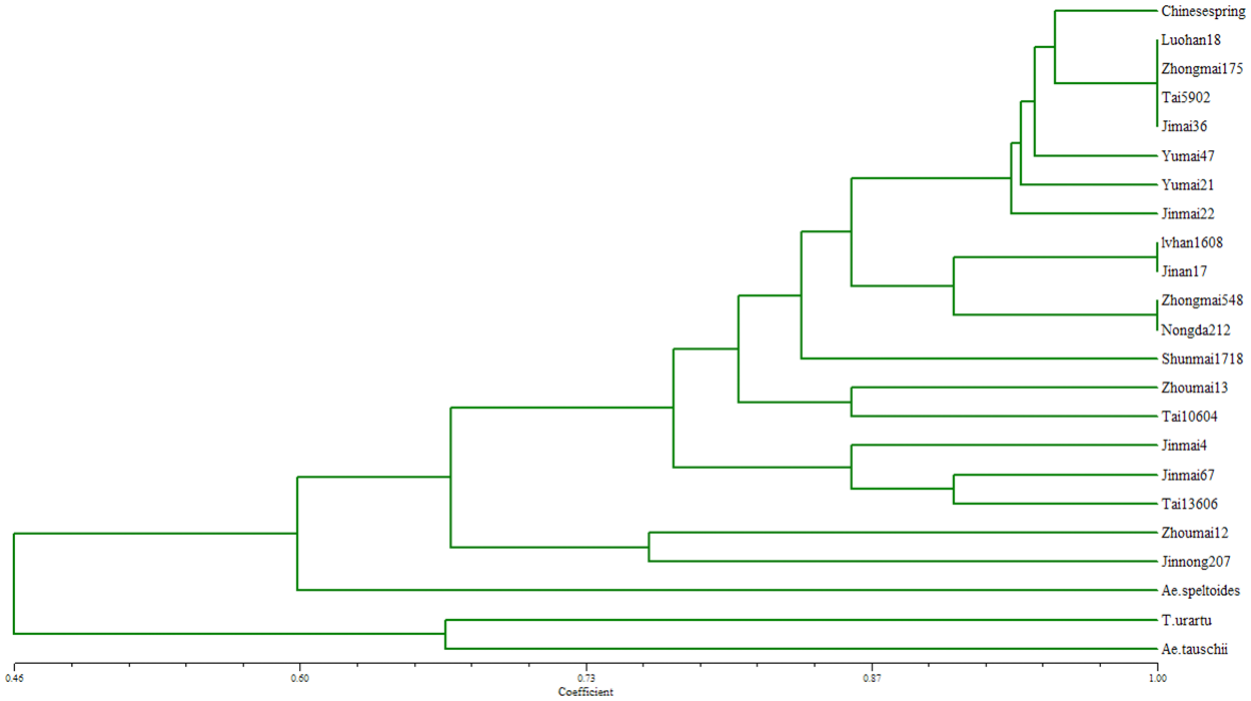
Phylogenetic relationship among 23 wheat cultivars was constructed using NTSYS cluster analysis.

A sub set of 45 monomorphic markers were selected randomly for validation in CSW genome (Table 6). Of the markers, 24 (53.3%) amplified one locus, which could be used for marker-assisted breeding in wheat. 8 (17.8% monomorphic SSR markers amplified multiple identical loci and 13 (28.9%) monomorphic SSR markers amplified no fragment from genome of CSW (Fig 4). These data will provide a solid base for our follow-up study. The 24 monomorphic SSR markers amplified one locus were used to analysis genetic relationship among 20 wheat cultivars and three species of its diploid ancestors. The phylogenetic relationship constructed in a dendrogram coefficients using Numerical Taxonomy System of Multivariate Programs (NTSYS) cluster analysis (Fig 5). At a similarity coefficient≥0.6, the largest group consisted of hexaploid wheat cultivars and the diploid ansestor of the B genome. The diploid ansestor of the A and D genomes were clustered into subgroups at similarity values of 0.67. Hexaploid wheat cultivars were clustered into two subgroups as well at the same values. Our results indicate that the monomorphic SSR markers had the ability to assess molecular diversity and potential for use in fingerprinting analysis.

**Table 6:** Characteristics of 45 SSR markers used in the present study.

ID of markers	Repeat motifs	Forward(5'-3')	Reverse(5'-3')	Tm(°C)
1	(GAA)7	AAACCCTTCTCCCATCTAGG	TAACCGCTAGGTTTAGTCCG	57.112
2	(TAG)22	GAGGTGACTGAGGCTCTTGT	CTACTACCACCACCACCACC	57.771
3	(TTC)6	ACGGTTGTCTCTTTAGGTGG	GACCGCAACAAAGTTACCTC	57.328
4	(CATCCT)4	CAATACTCAAGCTGGCAATG	GCATATGGTTTAGTCGGGTC	57.028
5	(CCA)6	ATCTCCCGCATGAGACCT	GAGCTACTGCTGCTCAAAGC	58.159
6	(CGGCGA)3	GAGCAAGTCATCAAGTCACG	GTGCAGTTTGGTCAAGTCAG	56.833
7	(CA)19	GTCAAAAGTTGTGGCATGTG	ATCAGAGCACCTCCAGAGTC	56.908
8	(GTC)7	CTGCACAACACTTATCGCTC	GAAACTCTGAAGCTGGTGGT	56.934
9	(CCG)6	AGTGCAAGACTAGGGTGGAG	GAAATGCGTTTGGTGGTTAG	58.147
10	(AAAAAG)3	TGGACATGCTATAGTGTGGG	GATGGGGATATAGCGAAATG	57.013
11	(CCCTCT)3	ATTCAGACACCGGAAGTGTT	TAGTCCTACATGGAAGGGGA	57.052
12	(TATT)7	CTTGTGAATCCCCAGTTTCT	GATGGAAATGGACAGAGAGG	57.063
13	(CCA)7	GCCGCGTACAGATAGAAGAT	GGTGTTGCTTACCTCTGCTT	57.078
14	(TTA)6	TAGAAAATGGGGTGTTCCAG	TAAGATAAGGACCTGGACGG	56.761
15	(CCG)7	CACACAGAGATCGAGTCAGG	AGCTAGGGTTTCCTCATGG	56.78
16	(GGA)7	CTGCAGATGATGAAGACGAG	TACCTTTTTACCCCAAGCAG	56.938
17	(TTC)8	GGGGAGGATATCTTGTTCCT	CCCAGCCAATCCTCTACTAA	56.914
18	(TG)11	ATCACATGTTCGTACAACGC	TGGACCCCTTAGTTGTGAGT	57.069
19	(GGA)6	GACCTAGCCATTTCTGTGCT	ATATCTTCCCCCTTTTGGAC	56.941
20	(AAAAAC)3	GGACAATGCTGGACGAGTA	AGGAACATAGGATGCGAACT	56.343
21	(AAAAG)4	GCTATTTTGTTTAGCTGCCC	GAGGGTGTGTAGGATTCGAG	57.162
22	(GA)9	GTTATGCGGTAGGAGCTTGT	CAGGTCCTATTTTGCTCGAT	56.909
23	(GA)9	CAGGAAGGTAGCTTCAAGGA	GAATGAATGAATGGGTGGAG	57.775
24	(TTG)8	ACCTCTAGCCTACCCCAACT	GACAGAGGCTCCTTCCACTA	57.032
25	(GTGCCC)3	CCGCAAGATAGTGTACATGC	TAGCAGCGATGTGATGTAGC	57.63
26	(TATAGA)3	AACAGAAAAACTGTAGCGCC	CTGGGCAAAAGAAGGTTAAG	56.672
27	(GTT)6	TGTGATGAAGCTCAAGGAGA	AGAGAGAGGAAGGATTCGGT	56.966
28	(CT)11	AGAGAAGGATGACTGGCAGA	TTAATCCCTTAGGTCCCTGC	58.136
29	(ACGGTG)3	TACATAGAGCTGGGATAGCG	CTGTTACCGTTGTATTCCCC	56.944
30	(TG)16	GAGCACATGTCGTACCTCAA	CGTCGGGTGAAGAATAACTT	56.822
31	(TG)16	ACTACCATTGGGTGGTAGGA	ACCTTTAGTACCGGTTGGTG	56.613
32	(TTCA)5	ATTCAATACCACCAGCCTTC	TGTAGTTTTAGCCCGTCACA	56.881
33	(TC)9	CAACTGCCCTATACCCAATC	GGATTCATGGAGAAACCTTG	57.017
34	(CAA)10	GTGCTGCAGTAGCAGTGTTT	AAGAGGAAGGAGATGCCTTT	57.075
35	(GGA)6	CCCTTTCCTGTTCCAAGTT	GAATAGTTTGGGTCGTTTCG	57.217
36	(CTTCTC)3	CCTAACAAAGCCCAGTTCTC	GAAGACTACATCCCACTGCC	57.158
37	(CT)10	TCTTCCTTCCTCGAACAAAC	ACATAACTGAAGGACGGGAA	57.113
38	(TAC)6	AGCTCTAGGAAATTCCCCTC	GAACAAACAGGCTCAGGTCT	56.934
39	(AAG)6	GACACTTGCCCTCTTGTTTT	GGGAGAGGTTGCTTGGTAT	56.613
40	(GGCTCC)3	ATGCTGGTACCTTTGAGAGC	ACGCCTCACAGAGAAAAATC	56.982
41	(ATAC)5	TCCTAGCACACACACACACA	GTAGCCCCACTGTGTAATCC	56.982
42	(AAAATA)3	GCGGCAGGATCTACTGTTAT	GGTACGTAGCTGATGATCCC	57.074
43	(TCATCG)3	CATCGTCATCATCTTCCTCA	ACGAAAGGATAGAACCACCA	57.113
44	(ATGTCG)4	TACCACTTGCACGACTTGAG	AAAAGAGAGCTCCAGCAAGA	57.018
45	(TC)14	GCGATCACATCTAGAACCCT	ACAAAGCTCGTGGTACATCA	56.753

## Discussion

### Comparative characterization of SSRs between CSW and other crops

We compared the density and distribution of SSRs in the Chinese spring genome with those in other plants. A total of 364,347 SSRs were identified from among 10,603,760 unique sequences in Chinese spring wheat, at a density of 36.68 SSR/Mb. This density is extremely low compared with the reported number of SSRs in the monocot species *Brachypodium* (191.3 SSR/Mb), sorghum (175.4 SSR/Mb) and rice (363.3 SSR/Mb), as well as *Arabidopsis* (418.6 SSR/Mb), *Medicago* (495.8 SSR/Mb), the chromosome 5D of *Aegilops tauschii* [19] and *Populus* (667.9 SSR/Mb). [20]. These data may reflect the differences in DNA levels between genomes [8]. The distribution and density of SSRs are highly variable, perhaps due to differences in search criteria and database mining tools [21]. Though differently-sized genomes may also contribute to affecting repetitiveness of microsatellites, the density of SSRs were not significantly related to genome size[22,23] Moreover, the density of SSRs is considerably higher in dicot species than in monocots. We found that dinucleotide repeats were the most frequent motifs on each chromosome in CSW on the whole genome, which was also reported for the sweet orange genome [24]. Biswas *et al*. calculated the g-SSR frequencies in 11 plant genomes and found that dinucleotide repeats were predominant in both monocot and dicot genomes [20]. This conclusion is consistent with the current results. Like the genomes of *Arabidopsis thaliana* and rice, CSW contained the most AG/TC dinucleotide repeats, followed by AC/GT and AT/AT repeats [25,26]. The basic SSR composition of Chinese spring gives priority to A and T in all types of repeats; for example, CG/CG occurred at the lowest density among dinucleotide repeat motifs. This is also the case for human, *Drosophila melanogaster* and other eukaryotic genomes [27]. Hong *et al*. [28] reported that GAA/TCC and AGA/TCT are the most frequent trinucleotide patterns in the *Solanaceae*, and CCG/CGG are the most abundant trinucleotide patterns in the coccolithophore *Emiliania huxleyi* [29]. Although the densities of trinucleotide repeat pattern are different among varies species, the most abundant patterns in CSW, *Arabidopsis* and *Brassicarapa* are identical; the greatest number of trinucleotide repeats comprise AAG/CTT [28]. Among tetra- to hexanucleotide repeats, AAAN, AAAAN and AAAAAN are much more common than other repeat motifs [28], which is also true for other plant genomes.

### Comparative characterization of SSRs between CSW and its related species

**Fig 6:**
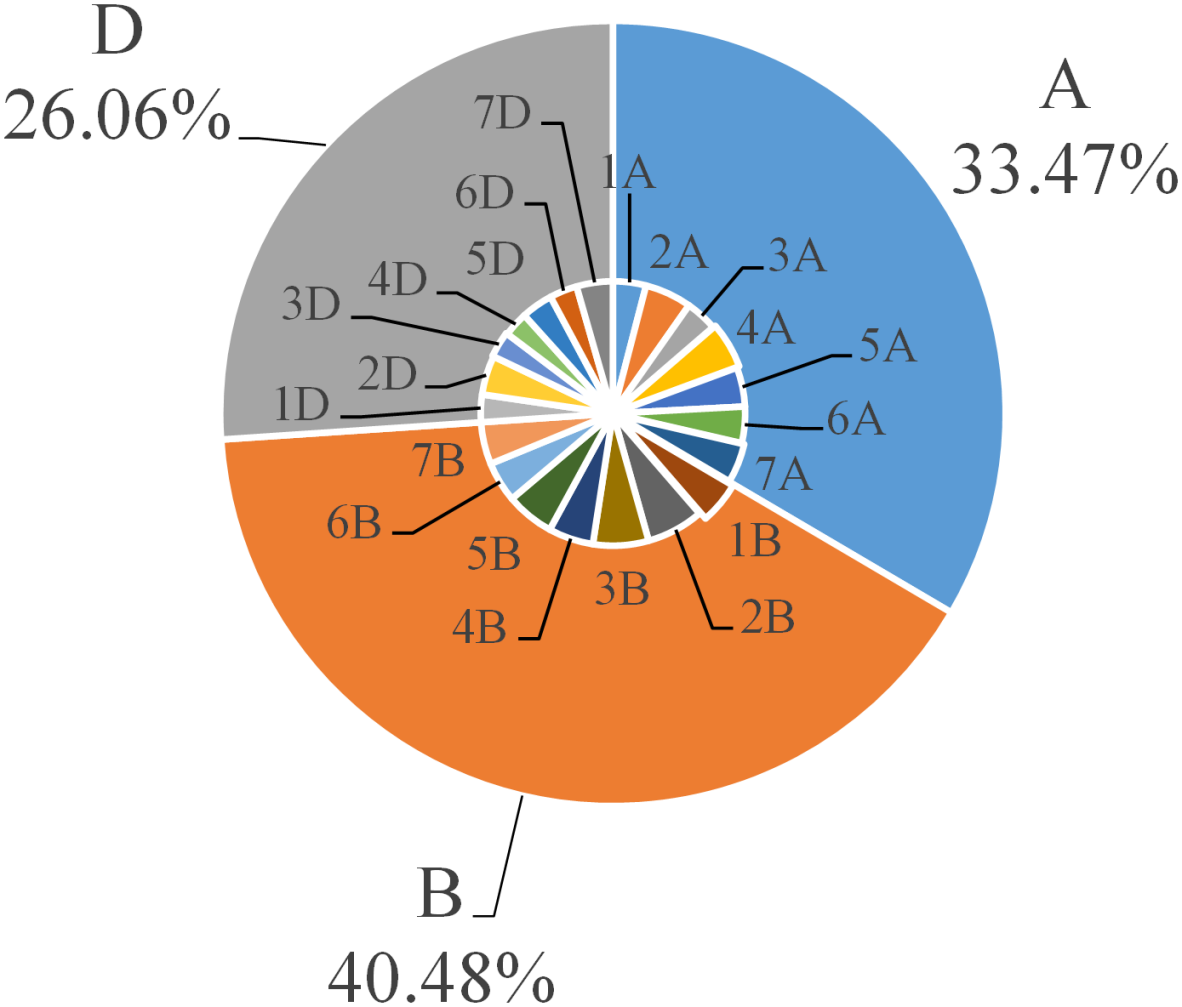
Distribution of g-SSRs in the Chinese spring homoeologous genomes.

**Fig 7:**
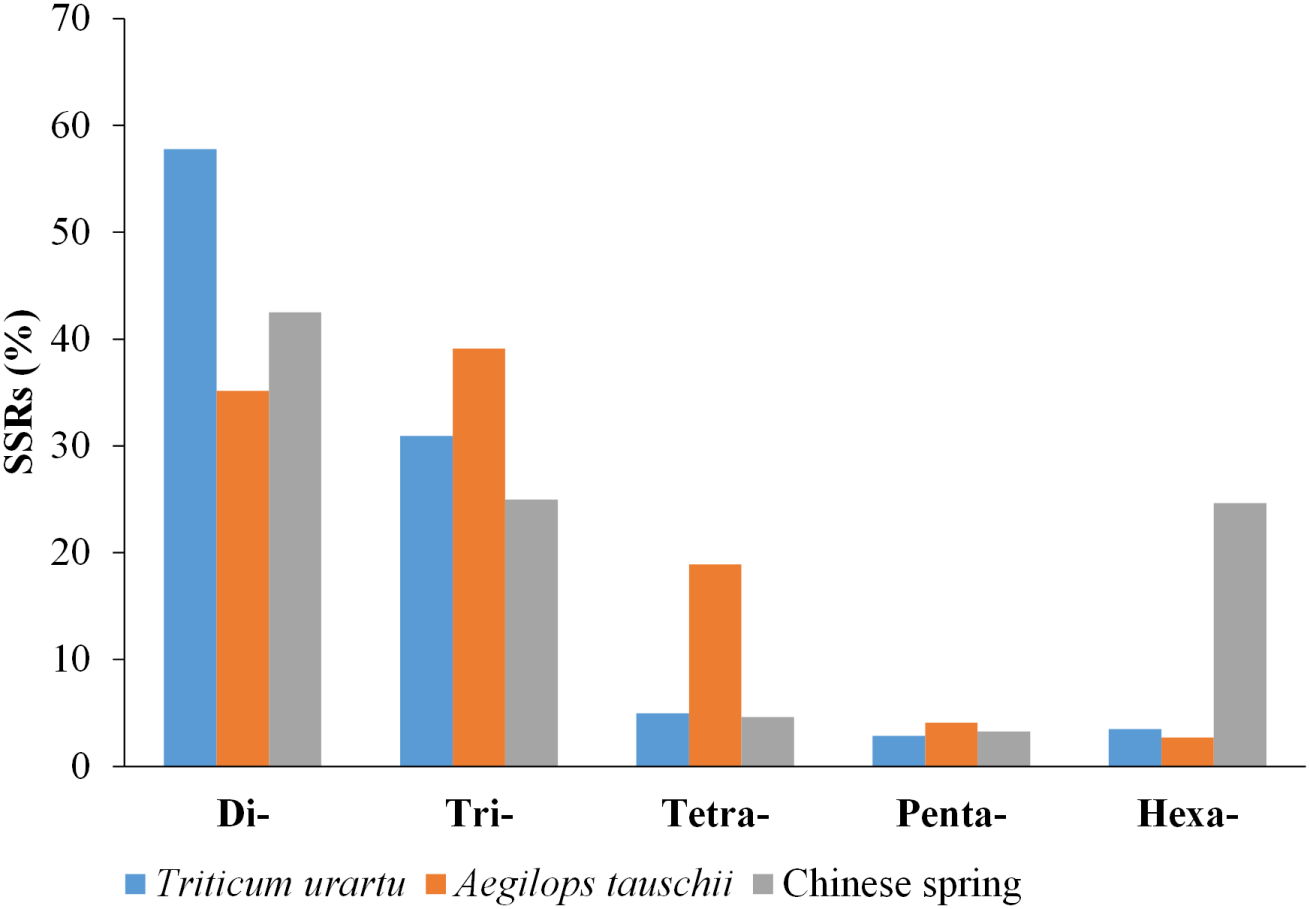
Comparison of SSRs among *Triticum urartu*, *Aegilops tauschii* and Chinese spring wheat.

The occurrence of SSRs in genomes mainly result from mutations during evolution, such as replication slippage, addition or removal of one or several repeat motifs. Therefore, the particular number and lengths of SSRs can serve as an index of genetic variation during the process of evolution. Chinese spring, an allohexaploid *Triticum aestivum* cultivar, contains three homoeologous genomes (A, B and D) [30]. The number of SSRs in the A, B and D genomes was 121,929, 147,479 and 94,939 respectively, with the B genome containing the largest number. These results suggest that there is the most variation in the B genome of CSW (Fig 6). This finding may at least partially explain why the draft genomes of the wheat A-genome progenitor *Triticum urartu* and D-genome progenitor *Aegilops tauschii* were sequenced, while there is currently no draft sequence for the B-genome progenitor [30,31]. We compared the g-SSR distribution in A-genome progenitor *Triticum urartu*, D-genome progenitor *Aegilops tauschii* and Chinese spring (Fig 7). In *Triticum urartu* andCSW, the dinucleotide repeat motifs were the predominant types (57.8% and 42.5%, respectively). By contrast, trinucleotide repeats were the most abundant motifs in *Aegilops tauschii* (39.13%). Trinucleotide repeats play an important role in specific selection against frameshift mutations in genetic regions. Trinucleotide repeats could refrain from selective pressures in coding regions owning to they had not generated frameshifts through expansion of triplet SSRs. But, non-triplet SSRs tended to face greater purifying selection with the frameshifts mutations. Therefore, the most percentage of trinucleotide repeats may related to high genic density in *Aegilops tauschii*[23,32,33]. Additionally, these variations of repeats are related to the different parameters used when mining SSRs of different species.

### Genome-wide SSR markers development and polymorphism analysis

As Cavagnaro *et al*. [34] noted, mononucleotide repeats are not suitable for marker development. Therefore, we only developed primers based on di- to hexanucleotide repeats. A total of 295,267 SSR markers were successfully designed, all of which were validated by re-PCR. Among the SSR markers, 70,564 (23.9%) were found to be monomorphic and 224,703 (76.1%) were found to be polymorphic. These monomorphic markers may serve as powerful tools for detecting sequence variation within a population of wheat and related species, examining the level of changes in genetic diversity and phylogenetic analyses [35]. Of the monomorphic markers, dinucleotide motifs (34.46%) were the most common, with chromosome 3B containing the most monomorphic markers, suggesting that the dinucleotide motifs on chromosome 3B may serve as better selectable markers for MAS in wheat. In addition, these markers represent an important genomic resource for use in many cereal crops and will benefit numerous genetic and genomic studies involving genetic diversity evaluation, population genetics, cloning functional genes related to agronomic and quality traits and comparative genomics in plants.

The validation of monomorphic SSR markers in the CSW genome was performed using 45 randomly selected monomorphic SSR markers. Eight amplified multiple loci. This may be a consequence of the fact that the full CSW genome sequence is not known and that there are large repetitive homologous sequences in non-homologous [6]. This can increase the frequency at which monomorphic makers amply multiple loci. Monomorphic makers which amplify one locus are more useful for phylogenetic analysis of wheat cultivars [36]. Analyses of phylogenetic relationship by using SSR markers could provide a better understanding of genetic background of wheat cultivars and become a foundation of the genetic improvement of wheat. The results of the present study indicated the monomorphic SSR markers used in this study might provide useful information for genetic improvement and germplasm conservation, evaluation and utilization in wheat. Monomorphic makers would play an important role in many aspects of wheat breeding, including in the identification of the genes responsible for desirable traits, and in the analysis of genetic relationships between and the diversity of wheat germplasm collections. Monomorphic markers should assist with improvements in wheat breeding[37].

## Supporting Information

S1 Table Summary of the frequency of different SSR repeat motif types in Chinese spring wheat. (DOCX)

S2 Table Distribution of different types of g-SSRs on Chinese spring chromosomes. (DOC)
