# Supplementary material for: Genome-Wide Analysis of Microsatellite Markers Based on Sequenced Database in Chinese Spring Wheat (Triticum aestivum L.)
Source: PLoS One. 2015 Nov 4;10(11):e0141540. doi: 10.1371/journal.pone.0141540 (PMC4633229; doi:10.1371/journal.pone.0141540)
Supplement: S1 Table — (DOCX) [file pone.0141540.s001.docx]

**Supporting Information**

**Table in S1 Table. Summary of the frequency of different SSR repeat motif types in Chinese spring wheat**

|  | **repeat** | **total** | **percentage(%)** |
| --- | --- | --- | --- |
| Di- | AG/CT | 91146 | 58.8369 |
|  | AC/GT | 44686 | 28.84587 |
|  | AT/AT | 18813 | 12.14424 |
|  | CG/CG | 268 | 0.173 |
| Tri- | AAG/CTT | 27688 | 30.43975 |
|  | AAC/GTT | 19222 | 21.13237 |
|  | AGG/CCT | 10284 | 11.30607 |
|  | AGT/ATC | 7730 | 8.498241 |
|  | ACT/ATG | 7564 | 8.315743 |
|  | AAT/ATT | 5429 | 5.968558 |
|  | ACC/GGT | 4115 | 4.523967 |
|  | CCG/CGG | 3294 | 3.621372 |
|  | AGC/CGT | 2848 | 3.131047 |
|  | ACG/CTG | 2786 | 3.062885 |
| Tetra- | AGAT/ATCT | 2872 | 17.07187 |
|  | AAAT/ATTT | 2098 | 12.47102 |
|  | ACAT/ATGT | 1878 | 11.16329 |
|  | ACGT/ATGC | 1873 | 11.13357 |
|  | AAAG/CTTT | 1431 | 8.506212 |
|  | AAGG/CCTT | 773 | 4.5949 |
|  | AGGG/CCCT | 676 | 4.018308 |
|  | ACGC/CGTG | 659 | 3.917256 |
|  | ACCT/ATGG | 635 | 3.774594 |
|  | AGGT/ATCC | 579 | 3.441717 |
|  | AAAC/GTTT | 564 | 3.352553 |
|  | AGCT/ATCG | 317 | 1.884325 |
|  | AATT/AATT | 266 | 1.581169 |
|  | AGCG/CGCT | 238 | 1.41473 |
|  | AACT/ATTG | 236 | 1.402841 |
|  | AATC/AGTT | 200 | 1.188849 |
|  | ACGG/CCTG | 180 | 1.069964 |
|  | AGGC/CCGT | 168 | 0.998633 |
|  | AAGC/CGTT | 161 | 0.957023 |
|  | ACTC/AGTG | 142 | 0.844083 |
|  | AACG/CTTG | 139 | 0.82625 |
|  | AGCC/CGGT | 114 | 0.677644 |
|  | AATG/ACTT | 114 | 0.677644 |
|  | ACAG/CTGT | 106 | 0.63009 |
|  | AAGT/ATTC | 97 | 0.576592 |
|  | AACC/GGTT | 94 | 0.558759 |
|  | ACCG/CTGG | 88 | 0.523093 |
|  | CCGG/CCGG | 48 | 0.285324 |
|  | ACTG/ACTG | 37 | 0.219937 |
|  | AGTC/AGTC | 32 | 0.190216 |
|  | CCCG/CGGG | 7 | 0.04161 |
|  | ACCC/GGGT | 1 | 0.005944 |
| Penta- | AAAAG/CTTTT | 1923 | 16.25803 |
|  | AAAAT/ATTTT | 1865 | 15.76767 |
|  | AAAAC/GTTTT | 535 | 4.523165 |
|  | AGAGG/CCTCT | 481 | 4.066622 |
|  | AGGGG/CCCCT | 388 | 3.280352 |
|  | AAGAG/CTCTT | 359 | 3.035171 |
|  | AAACC/GGTTT | 210 | 1.775448 |
|  | CCCGG/CCGGG | 166 | 1.403449 |
|  | CCGCG/CGCGG | 155 | 1.31045 |
|  | AAGGG/CCCTT | 152 | 1.285086 |
|  | AGCGG/CCTCG | 150 | 1.268177 |
|  | AAACT/ATTTG | 150 | 1.268177 |
|  | ACCGC/CGTGG | 139 | 1.175178 |
|  | AAATC/AGTTT | 135 | 1.141359 |
|  | ACCCC/GGGGT | 135 | 1.141359 |
|  | AGGCG/CCGCT | 130 | 1.099087 |
|  | ACGCC/CGGTG | 126 | 1.065269 |
|  | ACACC/GGTGT | 111 | 0.938451 |
|  | AATGC/ACGTT | 109 | 0.921542 |
|  | AACGT/ATTGC | 107 | 0.904633 |
|  | ACTCT/AGATG | 106 | 0.896179 |
|  | AGAGT/ATCTC | 106 | 0.896179 |
|  | AATAT/ATATT | 105 | 0.887724 |
|  | AAAGG/CCTTT | 104 | 0.87927 |
|  | ACTAG/ATCTG | 103 | 0.870815 |
|  | AGATC/AGTCT | 102 | 0.862361 |
|  | AGCCC/CGGGT | 97 | 0.820088 |
|  | AACCT/ATTGG | 96 | 0.811633 |
|  | ACACT/ATGTG | 87 | 0.735543 |
|  | AATCC/AGGTT | 86 | 0.727088 |
|  | AACCC/GGGTT | 85 | 0.718634 |
|  | AGAGC/CGTCT | 85 | 0.718634 |
|  | AATGG/ACCTT | 84 | 0.710179 |
|  | AGCCG/CGGCT | 84 | 0.710179 |
|  | AACAT/ATTGT | 83 | 0.701725 |
|  | AAGAT/ATTCT | 80 | 0.676361 |
|  | AATCT/AGATT | 79 | 0.667907 |
|  | AATGT/ACATT | 76 | 0.642543 |
|  | ACGAG/CTCTG | 76 | 0.642543 |
|  | ACCTC/AGTGG | 74 | 0.625634 |
|  | AATAC/ATGTT | 73 | 0.61718 |
|  | ACCCG/CTGGG | 70 | 0.591816 |
|  | ACGGG/CCCTG | 68 | 0.574907 |
|  | AATAG/ATCTT | 66 | 0.557998 |
|  | ACAGC/CGTGT | 65 | 0.549543 |
|  | ACTCC/AGGTG | 64 | 0.541089 |
|  | ACATC/AGTGT | 64 | 0.541089 |
|  | AGGGC/CCCGT | 62 | 0.52418 |
|  | AAAGT/ATTTC | 60 | 0.507271 |
|  | ACACG/CTGTG | 60 | 0.507271 |
|  | ACCGT/ATGGC | 60 | 0.507271 |
|  | AGGCC/CCGGT | 59 | 0.498816 |
|  | AAGGT/ATTCC | 54 | 0.456544 |
|  | AAATG/ACTTT | 53 | 0.448089 |
|  | ACCCT/ATGGG | 53 | 0.448089 |
|  | CCCCG/CGGGG | 52 | 0.439635 |
|  | AGCCT/ATCGG | 52 | 0.439635 |
|  | AAGTC/AGTTC | 51 | 0.43118 |
|  | AAATT/AATTT | 51 | 0.43118 |
|  | ACGGC/CCGTG | 51 | 0.43118 |
|  | AGGCT/ATCCG | 49 | 0.414271 |
|  | ACGGT/ATGCC | 48 | 0.405817 |
|  | ACTCG/AGCTG | 48 | 0.405817 |
|  | AGGTC/AGTCC | 46 | 0.388908 |
|  | ACCGG/CCTGG | 45 | 0.380453 |
|  | AAACG/CTTTG | 45 | 0.380453 |
|  | AACAC/GTGTT | 45 | 0.380453 |
|  | ACCTG/ACTGG | 43 | 0.363544 |
|  | ACTAT/ATATG | 43 | 0.363544 |
|  | AAAGC/CGTTT | 42 | 0.35509 |
|  | AACTG/ACTTG | 39 | 0.329726 |
|  | AGTAT/ATATC | 39 | 0.329726 |
|  | AGCTC/AGTCG | 39 | 0.329726 |
|  | ACGTG/ACTGC | 38 | 0.321272 |
|  | AGCAT/ATCGT | 37 | 0.312817 |
|  | ACGAT/ATGCT | 37 | 0.312817 |
|  | AGGGT/ATCCC | 36 | 0.304363 |
|  | ACGCG/CGCTG | 35 | 0.295908 |
|  | AAGCC/CGGTT | 35 | 0.295908 |
|  | AGCGC/CGCGT | 34 | 0.287454 |
|  | ACGTC/AGTGC | 34 | 0.287454 |
|  | AAGCT/ATTCG | 33 | 0.278999 |
|  | ACCAG/CTGGT | 33 | 0.278999 |
|  | AATCG/AGCTT | 31 | 0.26209 |
|  | AACCG/CTTGG | 28 | 0.236726 |
|  | AACGG/CCTTG | 27 | 0.228272 |
|  | ACAGT/ATGTC | 26 | 0.219817 |
|  | AACTT/AATTG | 26 | 0.219817 |
|  | AGGAT/ATCCT | 24 | 0.202908 |
|  | ACCAT/ATGGT | 24 | 0.202908 |
|  | ACATG/ACTGT | 24 | 0.202908 |
|  | AAGTG/ACTTC | 23 | 0.194454 |
|  | AACGC/CGTTG | 22 | 0.185999 |
|  | AAGTT/AATTC | 22 | 0.185999 |
|  | AAGAC/CTGTT | 20 | 0.16909 |
|  | ACAGG/CCTGT | 20 | 0.16909 |
|  | AAGGC/CCGTT | 18 | 0.152181 |
|  | AACAG/CTTGT | 17 | 0.143727 |
|  | AGCGT/ATCGC | 16 | 0.135272 |
|  | ACGCT/ATGCG | 12 | 0.101454 |
|  | AACTC/AGTTG | 8 | 0.067636 |
|  | AAGCG/CGCTT | 5 | 0.042273 |
| Hexa- | AAAATT/AATTTT | 7076 | 7.877715 |
|  | AAAAAG/CTTTTT | 6218 | 6.922503 |
|  | AGAGGG/CCCTCT | 5365 | 5.972858 |
|  | AAAAAT/ATTTTT | 3452 | 3.843114 |
|  | AAGAGG/CCTTCT | 3085 | 3.434532 |
|  | AAGGAG/CCTCTT | 3053 | 3.398907 |
|  | AGATAT/ATATCT | 2673 | 2.975853 |
|  | AAACTT/AATTTG | 2008 | 2.235508 |
|  | AAATTC/AAGTTT | 1822 | 2.028434 |
|  | ACCATC/AGTGGT | 1541 | 1.715596 |
|  | ACCACT/ATGGTG | 1498 | 1.667724 |
|  | AAAAAC/GTTTTT | 1404 | 1.563074 |
|  | AATGGG/ACCCTT | 896 | 0.997517 |
|  | AAGGGT/ATTCCC | 837 | 0.931833 |
|  | AGGGCG/CCCGCT | 801 | 0.891754 |
|  | AGAGAT/ATCTCT | 778 | 0.866148 |
|  | AGAGGT/ATCTCC | 773 | 0.860581 |
|  | AGCGGG/CCCTCG | 755 | 0.840542 |
|  | AAGAGC/CGTTCT | 739 | 0.822729 |
|  | AACCGC/CGTTGG | 729 | 0.811596 |
|  | ACCTCT/AGATGG | 720 | 0.801576 |
|  | AGGCGG/CCGCCT | 707 | 0.787104 |
|  | AACGAG/CTCTTG | 677 | 0.753705 |
|  | ACGAGG/CCTGCT | 676 | 0.752591 |
|  | ACTCCT/AGGATG | 673 | 0.749251 |
|  | AGCAGG/CCTCGT | 672 | 0.748138 |
|  | AACTCC/AGGTTG | 658 | 0.732552 |
|  | AGGAGT/ATCCTC | 618 | 0.68802 |
|  | AACCTC/AGTTGG | 607 | 0.675773 |
|  | ACACGC/CGTGTG | 592 | 0.659074 |
|  | AGGGGT/ATCCCC | 582 | 0.647941 |
|  | ACCTCC/AGGTGG | 566 | 0.630128 |
|  | AGGGGG/CCCCCT | 562 | 0.625675 |
|  | AACGCC/CGGTTG | 555 | 0.617882 |
|  | ACCCCT/ATGGGG | 552 | 0.614542 |
|  | AAAATG/ACTTTT | 550 | 0.612315 |
|  | AAGATC/AGTTCT | 540 | 0.601182 |
|  | AATACT/ATGATT | 527 | 0.586709 |
|  | AACTAG/ATCTTG | 524 | 0.58337 |
|  | ACCCCC/GGGGGT | 507 | 0.564443 |
|  | AAAAGT/ATTTTC | 504 | 0.561104 |
|  | AACAGC/CGTTGT | 497 | 0.55331 |
|  | AATCCC/AGGGTT | 487 | 0.542177 |
|  | AACGAC/CTGTTG | 482 | 0.536611 |
|  | ACCGCC/CGGTGG | 481 | 0.535498 |
|  | AGCGGC/CCGTCG | 478 | 0.532158 |
|  | AATCAT/AGTATT | 475 | 0.528818 |
|  | AACCCT/ATTGGG | 470 | 0.523251 |
|  | AAAAGG/CCTTTT | 463 | 0.515458 |
|  | ACGGCG/CCGCTG | 454 | 0.505438 |
|  | AGCTCG/AGCTCG | 412 | 0.45868 |
|  | ACCTAT/ATATGG | 392 | 0.436414 |
|  | AAAATC/AGTTTT | 385 | 0.428621 |
|  | ACCACG/CTGGTG | 360 | 0.400788 |
|  | ACGATG/ACTGCT | 358 | 0.398562 |
|  | CCGCGG/CCGGCG | 353 | 0.392995 |
|  | ACCAGC/CGTGGT | 340 | 0.378522 |
|  | AAAACT/ATTTTG | 337 | 0.375182 |
|  | AGGTAT/ATATCC | 329 | 0.366276 |
|  | ACGGAG/CCTCTG | 322 | 0.358483 |
|  | ACTAGT/ATCATG | 321 | 0.357369 |
|  | AGCAGT/ATCGTC | 320 | 0.356256 |
|  | AGAGGC/CCGTCT | 312 | 0.34735 |
|  | ACACTC/AGTGTG | 304 | 0.338443 |
|  | AAAGGT/ATTTCC | 291 | 0.32397 |
|  | AAATGG/ACCTTT | 291 | 0.32397 |
|  | ACATCT/AGATGT | 285 | 0.317291 |
|  | AAATAG/ATCTTT | 276 | 0.307271 |
|  | AAAGAG/CTCTTT | 273 | 0.303931 |
|  | AGCCGG/CCTCGG | 259 | 0.288345 |
|  | AAAGAT/ATTTCT | 258 | 0.287232 |
|  | AACAGT/ATTGTC | 256 | 0.285005 |
|  | ACGGGC/CCCGTG | 252 | 0.280552 |
|  | AGGCCG/CCGGCT | 245 | 0.272759 |
|  | AATGAC/ACTGTT | 238 | 0.264966 |
|  | ACATAT/ATATGT | 223 | 0.248266 |
|  | ACGCCG/CGGCTG | 215 | 0.23936 |
|  | AGCCGC/CGGCGT | 212 | 0.23602 |
|  | ACACCC/GGGTGT | 212 | 0.23602 |
|  | AGCCTC/AGTCGG | 211 | 0.234906 |
|  | AATCAG/AGTCTT | 211 | 0.234906 |
|  | ACTCCG/AGGCTG | 211 | 0.234906 |
|  | AAAGCC/CGGTTT | 209 | 0.23268 |
|  | AAGACT/ATTCTG | 207 | 0.230453 |
|  | AACAAG/CTTGTT | 205 | 0.228227 |
|  | AAGACG/CTGCTT | 200 | 0.22266 |
|  | AAGAAT/ATTCTT | 197 | 0.21932 |
|  | ACCTCG/AGCTGG | 195 | 0.217094 |
|  | AAGTAG/ATCTTC | 192 | 0.213754 |
|  | AAATAT/ATATTT | 191 | 0.21264 |
|  | AATGAT/ACTATT | 191 | 0.21264 |
|  | AATAGT/ATCATT | 190 | 0.211527 |
|  | ACAGAG/CTCTGT | 189 | 0.210414 |
|  | AAGCAG/CGTCTT | 189 | 0.210414 |
|  | ACTAGC/ATCGTG | 188 | 0.209301 |
|  | AAGGGG/CCCCTT | 188 | 0.209301 |
|  | AAACCG/CTTTGG | 176 | 0.195941 |
|  | AAACCC/GGGTTT | 176 | 0.195941 |
|  | AAGATG/ACTTCT | 175 | 0.194828 |
|  | ACGATC/AGTGCT | 173 | 0.192601 |
|  | AGCTCC/AGGTCG | 170 | 0.189261 |
|  | AAACAC/GTGTTT | 164 | 0.182581 |
|  | AAAGGC/CCGTTT | 164 | 0.182581 |
|  | AAGGGC/CCCGTT | 155 | 0.172562 |
|  | AGATCC/AGGTCT | 152 | 0.169222 |
|  | AAACGG/CCTTTG | 149 | 0.165882 |
|  | AAAACC/GGTTTT | 148 | 0.164768 |
|  | ACGGGG/CCCCTG | 147 | 0.163655 |
|  | ACCCGC/CGTGGG | 146 | 0.162542 |
|  | ACCCAT/ATGGGT | 146 | 0.162542 |
|  | AACGGG/CCCTTG | 143 | 0.159202 |
|  | AACATG/ACTTGT | 140 | 0.155862 |
|  | ACGGCC/CCGGTG | 140 | 0.155862 |
|  | ACCTAG/ATCTGG | 136 | 0.151409 |
|  | AGGGGC/CCCCGT | 135 | 0.150296 |
|  | ACAGTC/AGTGTC | 135 | 0.150296 |
|  | ACACTG/ACTGTG | 134 | 0.149182 |
|  | AATGGT/ACCATT | 134 | 0.149182 |
|  | AACCAG/CTTGGT | 134 | 0.149182 |
|  | AAAACG/CTTTTG | 133 | 0.148069 |
|  | ACCGGC/CCGTGG | 133 | 0.148069 |
|  | AGCCCC/CGGGGT | 132 | 0.146956 |
|  | AAATCC/AGGTTT | 130 | 0.144729 |
|  | AAGACC/CTGGTT | 130 | 0.144729 |
|  | ACTCTC/AGAGTG | 128 | 0.142502 |
|  | ACCCCG/CTGGGG | 124 | 0.138049 |
|  | ACCGGT/ATGGCC | 124 | 0.138049 |
|  | ACACAT/ATGTGT | 123 | 0.136936 |
|  | ACGCCC/CGGGTG | 121 | 0.134709 |
|  | AAAGTC/AGTTTC | 120 | 0.133596 |
|  | ACCTGC/ACGTGG | 116 | 0.129143 |
|  | CCCCCG/CGGGGG | 116 | 0.129143 |
|  | ACGTCC/AGGTGC | 115 | 0.12803 |
|  | AAACCT/ATTTGG | 115 | 0.12803 |
|  | ACATGC/ACGTGT | 115 | 0.12803 |
|  | ACGGTG/ACTGCC | 115 | 0.12803 |
|  | AAAGGG/CCCTTT | 114 | 0.126916 |
|  | AAATTT/AAATTT | 114 | 0.126916 |
|  | AAGTAC/ATGTTC | 112 | 0.12469 |
|  | AAAAGC/CGTTTT | 109 | 0.12135 |
|  | AGAGCG/CGCTCT | 109 | 0.12135 |
|  | AATGGC/ACCGTT | 108 | 0.120236 |
|  | AACACC/GGTTGT | 107 | 0.119123 |
|  | ACACCT/ATGTGG | 107 | 0.119123 |
|  | AACCAC/GGTGTT | 106 | 0.11801 |
|  | AAACTG/ACTTTG | 103 | 0.11467 |
|  | AACGGC/CCGTTG | 103 | 0.11467 |
|  | AACCCC/GGGGTT | 103 | 0.11467 |
|  | ACATCC/AGGTGT | 103 | 0.11467 |
|  | ACACGT/ATGTGC | 100 | 0.11133 |
|  | ACGAGT/ATGCTC | 100 | 0.11133 |
|  | AACGAT/ATTGCT | 100 | 0.11133 |
|  | AACGGT/ATTGCC | 98 | 0.109103 |
|  | AACCTG/ACTTGG | 98 | 0.109103 |
|  | AACTAC/ATGTTG | 98 | 0.109103 |
|  | ACCGTC/AGTGGC | 97 | 0.10799 |
|  | ACGTCG/AGCTGC | 97 | 0.10799 |
|  | CCCCGG/CCGGGG | 96 | 0.106877 |
|  | ACTCGT/AGCATG | 93 | 0.103537 |
|  | AGGCTC/AGTCCG | 93 | 0.103537 |
|  | AAAGTT/AATTTC | 93 | 0.103537 |
|  | AAATTG/AACTTT | 92 | 0.102424 |
|  | AAGGAC/CCTGTT | 90 | 0.100197 |
|  | ACCGAG/CTCTGG | 90 | 0.100197 |
|  | ACGTAT/ATATGC | 89 | 0.099084 |
|  | AAACAG/CTTTGT | 89 | 0.099084 |
|  | ACTCGG/AGCCTG | 88 | 0.09797 |
|  | AGAGCC/CGGTCT | 87 | 0.096857 |
|  | CCCGCG/CGCGGG | 85 | 0.094631 |
|  | AACACT/ATTGTG | 83 | 0.092404 |
|  | AGCGGT/ATCGCC | 83 | 0.092404 |
|  | AACATC/AGTTGT | 83 | 0.092404 |
|  | AAGTCC/AGGTTC | 82 | 0.091291 |
|  | AAACAT/ATTTGT | 81 | 0.090177 |
|  | AATATT/AATTAT | 81 | 0.090177 |
|  | AGCCGT/ATCGGC | 80 | 0.089064 |
|  | AACACG/CTTGTG | 80 | 0.089064 |
|  | ACTCCC/AGGGTG | 79 | 0.087951 |
|  | AACTCG/AGCTTG | 79 | 0.087951 |
|  | ACCGCG/CGCTGG | 79 | 0.087951 |
|  | ACGTCT/AGATGC | 79 | 0.087951 |
|  | ACGCGC/CGCGTG | 77 | 0.085724 |
|  | AGCCCG/CGGGCT | 77 | 0.085724 |
|  | AAGCAC/CGTGTT | 74 | 0.082384 |
|  | AAAGAC/CTGTTT | 74 | 0.082384 |
|  | ACGGCT/ATGCCG | 73 | 0.081271 |
|  | AAATAC/ATGTTT | 71 | 0.079044 |
|  | AACCAT/ATTGGT | 70 | 0.077931 |
|  | AATAGC/ATCGTT | 70 | 0.077931 |
|  | ACCGCT/ATGGCG | 69 | 0.076818 |
|  | ACGTAG/ATCTGC | 68 | 0.075704 |
|  | ACGCGT/ATGCGC | 68 | 0.075704 |
|  | ACGACT/ATGCTG | 67 | 0.074591 |
|  | AACAGG/CCTTGT | 67 | 0.074591 |
|  | AAGCTC/AGTTCG | 67 | 0.074591 |
|  | AAGGCG/CCGCTT | 66 | 0.073478 |
|  | AGCATC/AGTCGT | 65 | 0.072365 |
|  | AGCGCC/CGCGGT | 64 | 0.071251 |
|  | ACGCGG/CCTGCG | 64 | 0.071251 |
|  | AAGCCC/CGGGTT | 62 | 0.069025 |
|  | ACAGAT/ATGTCT | 61 | 0.067911 |
|  | AGTATC/AGTCAT | 60 | 0.066798 |
|  | ACCCTC/AGTGGG | 58 | 0.064571 |
|  | AATCAC/AGTGTT | 57 | 0.063458 |
|  | AGAGCT/ATCTCG | 57 | 0.063458 |
|  | ACACCG/CTGTGG | 57 | 0.063458 |
|  | ACGAGC/CGTGCT | 57 | 0.063458 |
|  | AATACC/ATGGTT | 56 | 0.062345 |
|  | AAGGTG/ACTTCC | 56 | 0.062345 |
|  | AGATCG/AGCTCT | 55 | 0.061232 |
|  | AACGTG/ACTTGC | 55 | 0.061232 |
|  | ACCCGG/CCTGGG | 55 | 0.061232 |
|  | ACGGGT/ATGCCC | 54 | 0.060118 |
|  | AAGGCC/CCGGTT | 53 | 0.059005 |
|  | AAGTAT/ATATTC | 53 | 0.059005 |
|  | AATCCT/AGGATT | 52 | 0.057892 |
|  | AACCTT/AATTGG | 51 | 0.056778 |
|  | ACTATG/ACTGAT | 51 | 0.056778 |
|  | AACCGG/CCTTGG | 51 | 0.056778 |
|  | AAGCGG/CCTTCG | 51 | 0.056778 |
|  | ACACAG/CTGTGT | 50 | 0.055665 |
|  | ACAGGT/ATGTCC | 49 | 0.054552 |
|  | AGGGCT/ATCCCG | 49 | 0.054552 |
|  | AAGCGT/ATTCGC | 48 | 0.053438 |
|  | ACCCGT/ATGGGC | 48 | 0.053438 |
|  | AAGATT/AATTCT | 47 | 0.052325 |
|  | ACAGCC/CGGTGT | 47 | 0.052325 |
|  | AAACGT/ATTTGC | 46 | 0.051212 |
|  | AATGCG/ACGCTT | 46 | 0.051212 |
|  | AGCGCG/CGCGCT | 46 | 0.051212 |
|  | AACATT/AATTGT | 45 | 0.050099 |
|  | AATATG/ACTTAT | 45 | 0.050099 |
|  | AGGCGC/CCGCGT | 45 | 0.050099 |
|  | AAAGTG/ACTTTC | 44 | 0.048985 |
|  | AACTGC/ACGTTG | 44 | 0.048985 |
|  | AAGTGC/ACGTTC | 44 | 0.048985 |
|  | AACCCG/CTTGGG | 44 | 0.048985 |
|  | AATGTG/ACACTT | 43 | 0.047872 |
|  | AAGTCG/AGCTTC | 42 | 0.046759 |
|  | AGGATC/AGTCCT | 42 | 0.046759 |
|  | ACATGG/ACCTGT | 42 | 0.046759 |
|  | AGGCCC/CCGGGT | 41 | 0.045645 |
|  | AAGCCG/CGGCTT | 41 | 0.045645 |
|  | AACTCT/AGATTG | 41 | 0.045645 |
|  | AACAAT/ATTGTT | 40 | 0.044532 |
|  | ACTAGG/ATCCTG | 39 | 0.043419 |
|  | AGCCCT/ATCGGG | 38 | 0.042305 |
|  | AACGTC/AGTTGC | 38 | 0.042305 |
|  | AAGTGG/ACCTTC | 38 | 0.042305 |
|  | ACATAG/ATCTGT | 38 | 0.042305 |
|  | AAATGC/ACGTTT | 38 | 0.042305 |
|  | AATCTT/AATTAG | 37 | 0.041192 |
|  | AATCTC/AGAGTT | 37 | 0.041192 |
|  | AGGGCC/CCCGGT | 37 | 0.041192 |
|  | ACCCTG/ACTGGG | 36 | 0.040079 |
|  | AATCTG/ACTTAG | 35 | 0.038966 |
|  | AACTTC/AAGTTG | 35 | 0.038966 |
|  | ACTCGC/AGCGTG | 35 | 0.038966 |
|  | ACAGGC/CCGTGT | 35 | 0.038966 |
|  | AAGTGT/ACATTC | 34 | 0.037852 |
|  | AGGGAT/ATCCCT | 34 | 0.037852 |
|  | AACCGT/ATTGGC | 34 | 0.037852 |
|  | ACCGTG/ACTGGC | 33 | 0.036739 |
|  | AATGCC/ACGGTT | 33 | 0.036739 |
|  | AGGGTC/AGTCCC | 32 | 0.035626 |
|  | ACCGGG/CCCTGG | 32 | 0.035626 |
|  | ACCCAG/CTGGGT | 30 | 0.033399 |
|  | ACACGG/CCTGTG | 30 | 0.033399 |
|  | ACGCTC/AGTGCG | 30 | 0.033399 |
|  | ACGCAG/CGTCTG | 29 | 0.032286 |
|  | ACGGTC/AGTGCC | 29 | 0.032286 |
|  | ACAGCG/CGCTGT | 28 | 0.031172 |
|  | AAGGTT/AATTCC | 28 | 0.031172 |
|  | AAGCTG/ACTTCG | 28 | 0.031172 |
|  | AACTGG/ACCTTG | 28 | 0.031172 |
|  | ACATGT/ACATGT | 27 | 0.030059 |
|  | AACGCT/ATTGCG | 27 | 0.030059 |
|  | ACTCTG/ACTGAG | 27 | 0.030059 |
|  | AAGTCT/AGATTC | 26 | 0.028946 |
|  | ACGCCT/ATGCGG | 26 | 0.028946 |
|  | AGAGTC/AGTCTC | 26 | 0.028946 |
|  | ACAGGG/CCCTGT | 26 | 0.028946 |
|  | AATCGC/AGCGTT | 25 | 0.027833 |
|  | ACCAGT/ATGGTC | 25 | 0.027833 |
|  | AAACTC/AGTTTG | 25 | 0.027833 |
|  | AGGTCC/AGGTCC | 25 | 0.027833 |
|  | AAGTTC/AAGTTC | 25 | 0.027833 |
|  | AATACG/ATGCTT | 25 | 0.027833 |
|  | AATAGG/ATCCTT | 24 | 0.026719 |
|  | AGCGTC/AGTCGC | 24 | 0.026719 |
|  | AATGTT/AATTAC | 24 | 0.026719 |
|  | AACTTG/AACTTG | 23 | 0.025606 |
|  | ACATCG/AGCTGT | 23 | 0.025606 |
|  | AGATCT/AGATCT | 23 | 0.025606 |
|  | AAGGTC/AGTTCC | 23 | 0.025606 |
|  | AAGCAT/ATTCGT | 22 | 0.024493 |
|  | ACGCTG/ACTGCG | 21 | 0.023379 |
|  | ACTCAT/AGTATG | 21 | 0.023379 |
|  | ACAGCT/ATGTCG | 20 | 0.022266 |
|  | AGCCAT/ATCGGT | 20 | 0.022266 |
|  | AAAGCT/ATTTCG | 20 | 0.022266 |
|  | AAGGAT/ATTCCT | 20 | 0.022266 |
|  | AATGAG/ACTCTT | 20 | 0.022266 |
|  | AAGAGT/ATTCTC | 19 | 0.021153 |
|  | ACCTGG/ACCTGG | 19 | 0.021153 |
|  | ACCAGG/CCTGGT | 19 | 0.021153 |
|  | AAATCG/AGCTTT | 19 | 0.021153 |
|  | AATCGT/AGCATT | 19 | 0.021153 |
|  | AAATCT/AGATTT | 18 | 0.020039 |
|  | ACCATG/ACTGGT | 18 | 0.020039 |
|  | AACTAT/ATATTG | 18 | 0.020039 |
|  | AAATGT/ACATTT | 18 | 0.020039 |
|  | ACAGTG/ACTGTC | 18 | 0.020039 |
|  | ACCGAT/ATGGCT | 17 | 0.018926 |
|  | ACTATC/AGTGAT | 17 | 0.018926 |
|  | AAAGCG/CGCTTT | 16 | 0.017813 |
|  | AACGTT/AATTGC | 16 | 0.017813 |
|  | AATATC/AGTTAT | 15 | 0.0167 |
|  | AATCGG/AGCCTT | 14 | 0.015586 |
|  | AGCTAT/ATATCG | 13 | 0.014473 |
|  | AAACGC/CGTTTG | 13 | 0.014473 |
|  | ACGTGC/ACGTGC | 12 | 0.01336 |
|  | AGGCGT/ATCCGC | 12 | 0.01336 |
|  | AACGCG/CGCTTG | 11 | 0.012246 |
|  | AATCCG/AGGCTT | 11 | 0.012246 |
|  | ACGGAT/ATGCCT | 11 | 0.012246 |
|  | AGGCAT/ATCCGT | 11 | 0.012246 |
|  | AAGGCT/ATTCCG | 10 | 0.011133 |
|  | ACGCAT/ATGCGT | 10 | 0.011133 |
|  | AGGCCT/ATCCGG | 9 | 0.01002 |
|  | AAGCGC/CGCGTT | 8 | 0.008906 |
|  | AATGCT/ACGATT | 8 | 0.008906 |
|  | ACTCAG/AGTCTG | 8 | 0.008906 |
|  | AGCGCT/ATCGCG | 8 | 0.008906 |
|  | AATGTC/ACAGTT | 7 | 0.007793 |
|  | AAGCCT/ATTCGG | 6 | 0.00668 |
|  | CCCGGG/CCCGGG | 6 | 0.00668 |
|  | AACTGT/ACATTG | 6 | 0.00668 |
|  | AAGCTT/AATTCG | 3 | 0.00334 |
|  | AGCGAT/ATCGCT | 1 | 0.001113 |
