# Supplementary material for: Genome-Wide Analysis of Microsatellite Markers Based on Sequenced Database in Chinese Spring Wheat (Triticum aestivum L.)
Source: PLoS One. 2015 Nov 4;10(11):e0141540. doi: 10.1371/journal.pone.0141540 (PMC4633229; doi:10.1371/journal.pone.0141540)
Supplement: S2 Table — (DOC) [file pone.0141540.s002.doc]

**Supporting Information**

**Table in S2 Table. Distribution of different types of g-SSRs on Chinese spring chromosomes**

|  | | | | | | | | |
| --- | --- | --- | --- | --- | --- | --- | --- | --- |
| **Repeats** | **Chr-1** | | **Chr-2** | **Chr-3** | **Chr-4** | **Chr-5** | **Chr-6** | **Chr-7** |
| AG/CT | 11687 | | 15389 | 13348 | 12778 | 12864 | 11939 | 13141 |
| AC/GT | 5628 | | 7756 | 6414 | 6372 | 6234 | 5663 | 6619 |
| AT/AT | 1578 | | 3390 | 2581 | 2167 | 2408 | 2834 | 3855 |
| CG/CG | 30 | | 52 | 27 | 56 | 35 | 32 | 36 |
| AAG/CTT | 3658 | | 4827 | 3779 | 4008 | 3905 | 3408 | 4103 |
| AAC/GTT | 2484 | | 3113 | 2807 | 2978 | 2908 | 2434 | 2498 |
| AGG/CCT | 1325 | | 1888 | 1413 | 1488 | 1678 | 1222 | 1270 |
| AGT/ATC | 1032 | | 1335 | 1093 | 1072 | 1099 | 1032 | 1067 |
| ACT/ATG | 993 | | 1320 | 1073 | 1007 | 1112 | 985 | 1074 |
| ACC/GGT | 558 | | 734 | 526 | 649 | 696 | 471 | 481 |
| AAT/ATT | 491 | | 1008 | 800 | 690 | 653 | 747 | 1040 |
| CCG/CGG | 485 | | 836 | 242 | 581 | 568 | 386 | 196 |
| AGC/CGT | 416 | | 532 | 330 | 409 | 474 | 368 | 319 |
| ACG/CTG | | 380 | 575 | 342 | 396 | 449 | 351 | 293 |
| AGAT/ATCT | | 321 | 440 | 412 | 365 | 314 | 343 | 677 |
| AAAT/ATTT | | 304 | 348 | 292 | 280 | 292 | 284 | 298 |
| ACGT/ATGC | | 285 | 278 | 224 | 291 | 296 | 256 | 243 |
| ACAT/ATGT | | 232 | 319 | 253 | 307 | 270 | 196 | 301 |
| AAAG/CTTT | | 180 | 285 | 195 | 178 | 257 | 141 | 195 |
| AGGG/CCCT | | 92 | 135 | 86 | 97 | 100 | 72 | 94 |
| AAGG/CCTT | | 86 | 159 | 93 | 120 | 112 | 82 | 121 |
| AAAC/GTTT | | 78 | 106 | 58 | 87 | 101 | 48 | 86 |
| ACCT/ATGG | | 78 | 107 | 87 | 113 | 90 | 70 | 90 |
| AGGT/ATCC | | 71 | 91 | 89 | 94 | 86 | 56 | 92 |
| ACGC/CGTG | | 70 | 111 | 107 | 78 | 118 | 85 | 90 |
| AGCG/CGCT | | 31 | 55 | 24 | 37 | 42 | 30 | 19 |
| AATC/AGTT | | 30 | 31 | 35 | 34 | 28 | 20 | 22 |
| AACT/ATTG | | 29 | 49 | 28 | 36 | 35 | 26 | 33 |
| AAGC/CGTT | | 26 | 42 | 17 | 17 | 26 | 19 | 14 |
| AGCT/ATCG | | 26 | 61 | 34 | 51 | 56 | 41 | 48 |
| AGGC/CCGT | | 25 | 31 | 25 | 23 | 33 | 21 | 10 |
| AATT/AATT | | 24 | 63 | 29 | 42 | 33 | 29 | 46 |
| ACTC/AGTG | | 20 | 19 | 16 | 18 | 27 | 21 | 21 |
| AACG/CTTG | | 19 | 40 | 18 | 22 | 14 | 14 | 12 |
| ACAG/CTGT | | 17 | 22 | 9 | 13 | 23 | 10 | 12 |
| ACGG/CCTG | | 14 | 27 | 25 | 38 | 35 | 23 | 18 |
| AGCC/CGGT | | 12 | 19 | 14 | 15 | 27 | 17 | 10 |
| AATG/ACTT | | 10 | 23 | 15 | 14 | 22 | 16 | 14 |
| ACCG/CTGG | | 8 | 23 | 12 | 12 | 14 | 9 | 10 |
| AACC/GGTT | | 7 | 22 | 14 | 15 | 17 | 8 | 11 |
| ACTG/ACTG | | 7 | 8 | 3 | 9 | 3 | 4 | 3 |
| CCGG/CCGG | | 7 | 13 | 5 | 4 | 7 | 5 | 7 |
| AAGT/ATTC | | 6 | 14 | 15 | 11 | 21 | 11 | 19 |
| AGTC/AGTC | | 5 | 7 | 2 | 5 | 5 | 4 | 4 |
| CCCG/CGGG | | 1 | 0 | 0 | 1 | 1 | 2 | 2 |
| AAAAG/CTTTT | | 242 | 353 | 255 | 268 | 301 | 247 | 257 |
| AAAAT/ATTTT | | 202 | 317 | 285 | 211 | 257 | 194 | 399 |
| AAAAC/GTTTT | | 69 | 105 | 68 | 86 | 60 | 69 | 78 |
| AGAGG/CCTCT | | 57 | 93 | 64 | 70 | 79 | 55 | 63 |
| AGGGG/CCCCT | | 51 | 91 | 56 | 51 | 60 | 40 | 39 |
| AAGAG/CTCTT | | 37 | 66 | 58 | 56 | 42 | 46 | 54 |
| AAACC/GGTTT | | 31 | 42 | 21 | 36 | 31 | 27 | 22 |
| AACCT/ATTGG | | 23 | 21 | 9 | 12 | 20 | 5 | 6 |
| AGCGG/CCTCG | | 22 | 41 | 14 | 18 | 28 | 14 | 13 |
| AGCCC/CGGGT | | 20 | 9 | 7 | 13 | 20 | 18 | 10 |
| ACGCC/CGGTG | | 19 | 19 | 17 | 21 | 17 | 16 | 17 |
| AAATC/AGTTT | | 18 | 23 | 24 | 13 | 22 | 18 | 17 |
| AACAT/ATTGT | | 18 | 7 | 15 | 12 | 13 | 7 | 11 |
| AATGT/ACATT | | 17 | 11 | 7 | 7 | 5 | 13 | 16 |
| ACCGC/CGTGG | | 17 | 34 | 14 | 23 | 16 | 10 | 25 |
| ACTCT/AGATG | | 17 | 18 | 9 | 21 | 13 | 18 | 10 |
| CCCGG/CCGGG | | 17 | 34 | 22 | 31 | 29 | 13 | 20 |
| CCGCG/CGCGG | | 17 | 33 | 17 | 32 | 25 | 11 | 20 |
| AAAGG/CCTTT | | 16 | 20 | 14 | 19 | 17 | 10 | 8 |
| AACGT/ATTGC | | 16 | 10 | 15 | 17 | 21 | 15 | 13 |
| AATGG/ACCTT | | 16 | 8 | 3 | 7 | 39 | 3 | 8 |
| AACCC/GGGTT | | 15 | 7 | 10 | 20 | 13 | 9 | 11 |
| AAGGG/CCCTT | | 14 | 30 | 25 | 20 | 23 | 24 | 16 |
| AATGC/ACGTT | | 14 | 24 | 11 | 15 | 18 | 15 | 12 |
| ACCCC/GGGGT | | 14 | 36 | 17 | 18 | 23 | 15 | 12 |
| AGGCG/CCGCT | | 14 | 28 | 19 | 27 | 20 | 12 | 10 |
| AATCC/AGGTT | | 13 | 17 | 8 | 17 | 14 | 8 | 9 |
| ACACC/GGTGT | | 12 | 22 | 19 | 16 | 13 | 12 | 17 |
| ACACT/ATGTG | | 12 | 10 | 10 | 15 | 15 | 8 | 17 |
| ACGGG/CCCTG | | 12 | 14 | 6 | 11 | 7 | 8 | 10 |
| AGAGT/ATCTC | | 12 | 11 | 11 | 24 | 28 | 9 | 11 |
| AGATC/AGTCT | | 12 | 15 | 12 | 24 | 15 | 13 | 11 |
| AGCCG/CGGCT | | 12 | 19 | 9 | 13 | 10 | 14 | 7 |
| AAGAT/ATTCT | | 11 | 10 | 11 | 5 | 14 | 18 | 11 |
| AATAC/ATGTT | | 11 | 14 | 9 | 12 | 10 | 10 | 7 |
| ACCCG/CTGGG | | 11 | 15 | 6 | 10 | 7 | 13 | 8 |
| ACCGG/CCTGG | | 11 | 9 | 2 | 9 | 7 | 1 | 6 |
| ACCTC/AGTGG | | 11 | 9 | 11 | 16 | 18 | 5 | 4 |
| ACTAG/ATCTG | | 11 | 21 | 15 | 15 | 17 | 15 | 9 |
| AAATG/ACTTT | | 10 | 11 | 5 | 8 | 11 | 4 | 4 |
| AATAT/ATATT | | 10 | 17 | 16 | 13 | 5 | 25 | 19 |
| ACTCC/AGGTG | | 10 | 10 | 14 | 6 | 11 | 6 | 7 |
| AAAGT/ATTTC | | 9 | 16 | 6 | 6 | 8 | 5 | 10 |
| AAGTC/AGTTC | | 8 | 11 | 6 | 7 | 7 | 6 | 6 |
| AATAG/ATCTT | | 8 | 11 | 11 | 8 | 11 | 10 | 7 |
| ACACG/CTGTG | | 8 | 9 | 8 | 10 | 12 | 8 | 5 |
| ACAGC/CGTGT | | 8 | 9 | 11 | 16 | 10 | 4 | 7 |
| ACATC/AGTGT | | 8 | 13 | 7 | 10 | 11 | 6 | 9 |
| AAGCT/ATTCG | | 7 | 5 | 10 | 5 | 3 | 3 | 0 |
| AAGTG/ACTTC | | 7 | 4 | 2 | 2 | 2 | 1 | 5 |
| ACCAG/CTGGT | | 7 | 6 | 2 | 7 | 5 | 3 | 3 |
| AAACG/CTTTG | | 6 | 6 | 6 | 7 | 11 | 4 | 5 |
| AACTG/ACTTG | | 6 | 4 | 8 | 0 | 8 | 8 | 5 |
| AAGGT/ATTCC | | 6 | 6 | 11 | 5 | 16 | 3 | 7 |
| AATCT/AGATT | | 6 | 14 | 15 | 11 | 7 | 7 | 19 |
| ACCGT/ATGGC | | 6 | 12 | 9 | 7 | 11 | 9 | 6 |
| ACGAG/CTCTG | | 6 | 18 | 10 | 13 | 16 | 4 | 9 |
| ACGCG/CGCTG | | 6 | 6 | 6 | 3 | 8 | 1 | 5 |
| AGAGC/CGTCT | | 6 | 16 | 13 | 12 | 18 | 10 | 10 |
| AGCAT/ATCGT | | 6 | 14 | 4 | 5 | 3 | 1 | 4 |
| AGCGC/CGCGT | | 6 | 6 | 4 | 3 | 5 | 5 | 5 |
| AGGCC/CCGGT | | 6 | 15 | 9 | 9 | 12 | 4 | 4 |
| AGGTC/AGTCC | | 6 | 9 | 5 | 4 | 9 | 7 | 6 |
| CCCCG/CGGGG | | 6 | 14 | 7 | 11 | 8 | 2 | 4 |
| AAACT/ATTTG | | 5 | 40 | 21 | 27 | 21 | 19 | 17 |
| AACAC/GTGTT | | 5 | 7 | 9 | 5 | 6 | 3 | 10 |
| AACCG/CTTGG | | 5 | 7 | 4 | 4 | 2 | 2 | 4 |
| AACGG/CCTTG | | 5 | 3 | 5 | 5 | 2 | 5 | 2 |
| AAGGC/CCGTT | | 5 | 1 | 1 | 0 | 6 | 4 | 1 |
| ACGGT/ATGCC | | 5 | 9 | 9 | 5 | 11 | 4 | 5 |
| ACGTG/ACTGC | | 5 | 7 | 5 | 7 | 6 | 7 | 1 |
| AGGAT/ATCCT | | 5 | 3 | 2 | 4 | 0 | 4 | 6 |
| AGGGT/ATCCC | | 5 | 5 | 2 | 8 | 5 | 4 | 7 |
| AACAG/CTTGT | | 4 | 1 | 1 | 4 | 3 | 0 | 4 |
| AAATT/AATTT | | 4 | 14 | 10 | 6 | 4 | 3 | 10 |
| ACCAT/ATGGT | | 4 | 5 | 3 | 1 | 3 | 1 | 7 |
| ACCTG/ACTGG | | 4 | 7 | 5 | 9 | 7 | 4 | 7 |
| ACGAT/ATGCT | | 4 | 8 | 6 | 6 | 6 | 5 | 2 |
| ACGGC/CCGTG | | 4 | 11 | 13 | 5 | 7 | 2 | 9 |
| ACTAT/ATATG | | 4 | 11 | 12 | 7 | 5 | 0 | 4 |
| AGCCT/ATCGG | | 4 | 8 | 6 | 4 | 12 | 6 | 12 |
| AGGCT/ATCCG | | 4 | 8 | 5 | 8 | 9 | 5 | 10 |
| AGGGC/CCCGT | | 4 | 9 | 14 | 11 | 13 | 5 | 6 |
| AAAGC/CGTTT | | 3 | 7 | 3 | 5 | 9 | 7 | 8 |
| AACGC/CGTTG | | 3 | 4 | 2 | 4 | 4 | 3 | 2 |
| AAGCG/CGCTT | | 3 | 0 | 0 | 0 | 1 | 0 | 1 |
| AAGCC/CGGTT | | 3 | 6 | 3 | 13 | 5 | 2 | 3 |
| AAGTT/AATTC | | 3 | 5 | 3 | 5 | 3 | 1 | 2 |
| ACAGG/CCTGT | | 3 | 2 | 4 | 1 | 7 | 1 | 2 |
| ACAGT/ATGTC | | 3 | 4 | 4 | 4 | 5 | 3 | 3 |
| AGTAT/ATATC | | 3 | 5 | 8 | 6 | 5 | 4 | 8 |
| ACCCT/ATGGG | | 2 | 6 | 5 | 12 | 10 | 10 | 8 |
| ACTCG/AGCTG | | 2 | 10 | 10 | 6 | 7 | 4 | 9 |
| AGCGT/ATCGC | | 2 | 3 | 2 | 1 | 5 | 1 | 2 |
| AGCTC/AGTCG | | 2 | 8 | 10 | 4 | 4 | 5 | 6 |
| AACTT/AATTG | | 1 | 6 | 5 | 5 | 3 | 3 | 3 |
| AATCG/AGCTT | | 1 | 6 | 6 | 4 | 8 | 2 | 4 |
| ACATG/ACTGT | | 1 | 5 | 4 | 1 | 1 | 9 | 3 |
| ACGCT/ATGCG | | 1 | 4 | 1 | 1 | 1 | 1 | 3 |
| ACGTC/AGTGC | | 1 | 11 | 2 | 4 | 8 | 7 | 1 |
| AAAATT/AATTTT | | 978 | 952 | 977 | 636 | 1404 | 972 | 1157 |
| AAAAAG/CTTTTT | | 740 | 1062 | 876 | 932 | 821 | 833 | 954 |
| AGAGGG/CCCTCT | | 702 | 989 | 644 | 805 | 831 | 676 | 718 |
| AAGAGG/CCTTCT | | 421 | 504 | 428 | 492 | 444 | 398 | 398 |
| AAGGAG/CCTCTT | | 412 | 517 | 423 | 450 | 445 | 389 | 417 |
| AAAAAT/ATTTTT | | 388 | 649 | 464 | 484 | 470 | 449 | 548 |
| AGATAT/ATATCT | | 325 | 424 | 416 | 306 | 344 | 321 | 537 |
| AAACTT/AATTTG | | 255 | 360 | 257 | 336 | 268 | 263 | 269 |
| AAATTC/AAGTTT | | 220 | 297 | 220 | 311 | 257 | 241 | 276 |
| ACCATC/AGTGGT | | 176 | 281 | 218 | 236 | 238 | 192 | 200 |
| ACCACT/ATGGTG | | 175 | 274 | 197 | 233 | 223 | 175 | 221 |
| AAAAAC/GTTTTT | | 168 | 263 | 191 | 223 | 201 | 155 | 203 |
| AATGGG/ACCCTT | | 122 | 137 | 126 | 139 | 146 | 124 | 102 |
| AGCGGG/CCCTCG | | 122 | 105 | 101 | 115 | 121 | 96 | 95 |
| AAGGGT/ATTCCC | | 118 | 148 | 108 | 125 | 112 | 124 | 102 |
| AGGGCG/CCCGCT | | 112 | 143 | 108 | 129 | 107 | 106 | 96 |
| AAGAGC/CGTTCT | | 103 | 138 | 71 | 118 | 101 | 110 | 98 |
| AGGCGG/CCGCCT | | 103 | 134 | 74 | 120 | 113 | 80 | 83 |
| ACCTCC/AGGTGG | | 102 | 96 | 64 | 82 | 104 | 58 | 60 |
| AGAGGT/ATCTCC | | 102 | 139 | 115 | 141 | 101 | 95 | 80 |
| ACTCCT/AGGATG | | 101 | 117 | 84 | 97 | 92 | 86 | 96 |
| AGGAGT/ATCCTC | | 98 | 104 | 85 | 75 | 95 | 75 | 86 |
| AACTCC/AGGTTG | | 95 | 161 | 83 | 77 | 94 | 75 | 73 |
| ACCTCT/AGATGG | | 92 | 119 | 96 | 111 | 118 | 94 | 90 |
| AGAGAT/ATCTCT | | 92 | 161 | 108 | 108 | 100 | 97 | 112 |
| AACGCC/CGGTTG | | 91 | 96 | 65 | 73 | 66 | 92 | 72 |
| ACGAGG/CCTGCT | | 91 | 136 | 75 | 113 | 104 | 69 | 88 |
| AACCGC/CGTTGG | | 90 | 127 | 93 | 117 | 92 | 95 | 115 |
| ACACGC/CGTGTG | | 89 | 104 | 70 | 88 | 75 | 72 | 94 |
| AACCTC/AGTTGG | | 83 | 106 | 79 | 72 | 102 | 89 | 76 |
| AACGAG/CTCTTG | | 82 | 132 | 87 | 117 | 70 | 109 | 80 |
| ACCGCC/CGGTGG | | 82 | 97 | 51 | 65 | 100 | 53 | 33 |
| AGCAGG/CCTCGT | | 81 | 128 | 73 | 122 | 92 | 84 | 92 |
| AATACT/ATGATT | | 77 | 80 | 68 | 68 | 62 | 74 | 98 |
| AATCAT/AGTATT | | 76 | 61 | 63 | 66 | 71 | 57 | 81 |
| AGGGGG/CCCCCT | | 74 | 112 | 69 | 71 | 93 | 62 | 81 |
| AGGGGT/ATCCCC | | 74 | 107 | 58 | 85 | 83 | 75 | 100 |
| AAAATG/ACTTTT | | 72 | 88 | 76 | 66 | 97 | 72 | 79 |
| AAAAGG/CCTTTT | | 66 | 82 | 62 | 54 | 59 | 62 | 78 |
| AACTAG/ATCTTG | | 65 | 102 | 92 | 70 | 86 | 47 | 62 |
| AATCCC/AGGGTT | | 64 | 76 | 58 | 68 | 84 | 55 | 82 |
| ACCCCC/GGGGGT | | 64 | 85 | 82 | 109 | 66 | 53 | 48 |
| AAAAGT/ATTTTC | | 62 | 78 | 73 | 57 | 92 | 74 | 68 |
| ACCCCT/ATGGGG | | 62 | 105 | 55 | 94 | 78 | 86 | 72 |
| AGCGGC/CCGTCG | | 61 | 109 | 36 | 88 | 67 | 75 | 42 |
| ACGATG/ACTGCT | | 58 | 65 | 64 | 37 | 47 | 49 | 38 |
| AAGATC/AGTTCT | | 57 | 95 | 86 | 78 | 71 | 67 | 86 |
| ACGGAG/CCTCTG | | 57 | 53 | 48 | 45 | 38 | 38 | 43 |
| ACGGCG/CCGCTG | | 57 | 91 | 36 | 95 | 78 | 64 | 33 |
| AGCTCG/AGCTCG | | 57 | 70 | 49 | 64 | 55 | 52 | 65 |
| ACCACG/CTGGTG | | 52 | 54 | 51 | 60 | 55 | 46 | 42 |
| AACCCT/ATTGGG | | 49 | 80 | 69 | 67 | 67 | 66 | 72 |
| ACTAGT/ATCATG | | 47 | 52 | 42 | 52 | 36 | 39 | 53 |
| CCGCGG/CCGGCG | | 45 | 69 | 27 | 68 | 70 | 47 | 27 |
| AAAGGT/ATTTCC | | 43 | 47 | 35 | 39 | 37 | 47 | 43 |
| ACCTAT/ATATGG | | 43 | 72 | 64 | 60 | 38 | 44 | 71 |
| AGAGGC/CCGTCT | | 41 | 53 | 37 | 47 | 54 | 46 | 34 |
| ACCAGC/CGTGGT | | 40 | 49 | 44 | 63 | 55 | 37 | 52 |
| AGGTAT/ATATCC | | 40 | 63 | 43 | 50 | 43 | 44 | 46 |
| AACAGC/CGTTGT | | 39 | 73 | 120 | 64 | 96 | 56 | 49 |
| AAAACT/ATTTTG | | 38 | 74 | 40 | 46 | 61 | 26 | 52 |
| AAATAG/ATCTTT | | 37 | 53 | 30 | 26 | 31 | 45 | 54 |
| AGCCGC/CGGCGT | | 36 | 41 | 19 | 31 | 37 | 35 | 13 |
| AAATGG/ACCTTT | | 34 | 54 | 35 | 38 | 43 | 39 | 48 |
| ACATCT/AGATGT | | 34 | 51 | 39 | 60 | 38 | 33 | 30 |
| AAAATC/AGTTTT | | 33 | 79 | 57 | 63 | 37 | 42 | 74 |
| AGCAGT/ATCGTC | | 33 | 51 | 42 | 48 | 72 | 35 | 39 |
| AATGAC/ACTGTT | | 32 | 40 | 32 | 39 | 35 | 25 | 35 |
| ACGGGC/CCCGTG | | 32 | 60 | 25 | 37 | 29 | 30 | 39 |
| AAAGAG/CTCTTT | | 31 | 47 | 34 | 62 | 43 | 30 | 26 |
| AAAGAT/ATTTCT | | 31 | 50 | 36 | 32 | 35 | 19 | 55 |
| AAATAT/ATATTT | | 31 | 32 | 23 | 20 | 10 | 32 | 43 |
| AACAAG/CTTGTT | | 31 | 34 | 22 | 31 | 28 | 35 | 24 |
| ACTAGC/ATCGTG | | 31 | 31 | 27 | 21 | 24 | 26 | 28 |
| AGCCTC/AGTCGG | | 30 | 24 | 31 | 34 | 34 | 25 | 33 |
| AACGAC/CTGTTG | | 29 | 68 | 76 | 72 | 148 | 51 | 38 |
| AATCAG/AGTCTT | | 29 | 25 | 31 | 29 | 48 | 28 | 21 |
| AACAGT/ATTGTC | | 28 | 51 | 35 | 33 | 43 | 33 | 33 |
| AAGAAT/ATTCTT | | 28 | 35 | 35 | 21 | 32 | 21 | 25 |
| ACACTC/AGTGTG | | 28 | 64 | 44 | 35 | 46 | 34 | 53 |
| AGCCGG/CCTCGG | | 28 | 55 | 30 | 52 | 48 | 20 | 26 |
| AAACGG/CCTTTG | | 27 | 21 | 24 | 12 | 25 | 23 | 17 |
| AGGCCG/CCGGCT | | 27 | 41 | 29 | 42 | 56 | 26 | 24 |
| AAACCG/CTTTGG | | 26 | 33 | 21 | 19 | 34 | 24 | 19 |
| ACACTG/ACTGTG | | 26 | 22 | 23 | 12 | 20 | 21 | 10 |
| ACGCCG/CGGCTG | | 26 | 53 | 18 | 33 | 38 | 35 | 12 |
| ACTCCG/AGGCTG | | 26 | 37 | 39 | 25 | 38 | 27 | 19 |
| AAAACG/CTTTTG | | 25 | 24 | 18 | 19 | 14 | 15 | 18 |
| ACACCC/GGGTGT | | 25 | 45 | 33 | 25 | 32 | 24 | 28 |
| AAACAC/GTGTTT | | 24 | 36 | 24 | 25 | 16 | 16 | 23 |
| AAAGCC/CGGTTT | | 24 | 33 | 33 | 30 | 44 | 24 | 21 |
| AAAGGC/CCGTTT | | 24 | 22 | 28 | 27 | 27 | 21 | 15 |
| ACAGAG/CTCTGT | | 24 | 44 | 29 | 19 | 32 | 23 | 18 |
| ACATAT/ATATGT | | 24 | 34 | 34 | 37 | 39 | 23 | 32 |
| AGGGGC/CCCCGT | | 24 | 19 | 16 | 17 | 26 | 15 | 18 |
| AAGACG/CTGCTT | | 23 | 37 | 33 | 30 | 34 | 17 | 26 |
| AAGACT/ATTCTG | | 23 | 32 | 24 | 33 | 32 | 32 | 31 |
| AATGGT/ACCATT | | 23 | 19 | 18 | 23 | 20 | 19 | 12 |
| AACATG/ACTTGT | | 22 | 29 | 17 | 15 | 23 | 14 | 20 |
| AAGCAG/CGTCTT | | 22 | 39 | 26 | 33 | 33 | 16 | 20 |
| AAGTAG/ATCTTC | | 22 | 29 | 28 | 23 | 26 | 34 | 30 |
| AATGGC/ACCGTT | | 22 | 12 | 8 | 27 | 15 | 8 | 16 |
| ACCTCG/AGCTGG | | 21 | 39 | 28 | 29 | 37 | 21 | 20 |
| AAACTG/ACTTTG | | 20 | 13 | 15 | 13 | 16 | 13 | 13 |
| AAGATG/ACTTCT | | 20 | 30 | 30 | 23 | 28 | 19 | 25 |
| AAGGGC/CCCGTT | | 20 | 36 | 22 | 27 | 23 | 8 | 19 |
| AATAGT/ATCATT | | 20 | 34 | 25 | 35 | 24 | 22 | 30 |
| ACCCGC/CGTGGG | | 20 | 38 | 25 | 18 | 17 | 12 | 16 |
| AAATCC/AGGTTT | | 19 | 21 | 16 | 21 | 23 | 10 | 20 |
| ACGTCC/AGGTGC | | 19 | 22 | 11 | 24 | 16 | 10 | 13 |
| ACTCTC/AGAGTG | | 19 | 28 | 19 | 14 | 20 | 14 | 14 |
| AGCCCC/CGGGGT | | 19 | 21 | 22 | 21 | 29 | 9 | 11 |
| AGCCGT/ATCGGC | | 19 | 13 | 6 | 11 | 11 | 11 | 9 |
| AAACAT/ATTTGT | | 18 | 15 | 8 | 10 | 9 | 8 | 13 |
| AAAGGG/CCCTTT | | 18 | 12 | 14 | 21 | 18 | 10 | 21 |
| AACCAG/CTTGGT | | 18 | 26 | 17 | 24 | 20 | 13 | 16 |
| ACGTAT/ATATGC | | 18 | 17 | 10 | 16 | 10 | 9 | 9 |
| AGCTCC/AGGTCG | | 18 | 47 | 18 | 22 | 27 | 17 | 21 |
| AAAAGC/CGTTTT | | 17 | 24 | 8 | 13 | 23 | 13 | 11 |
| AAACCC/GGGTTT | | 17 | 27 | 26 | 26 | 25 | 19 | 36 |
| AAACCT/ATTTGG | | 17 | 21 | 14 | 14 | 21 | 17 | 11 |
| AACGGT/ATTGCC | | 17 | 13 | 16 | 17 | 11 | 13 | 11 |
| AAGACC/CTGGTT | | 17 | 22 | 22 | 23 | 15 | 10 | 21 |
| AAGTAC/ATGTTC | | 17 | 18 | 8 | 16 | 26 | 11 | 16 |
| ACCCAT/ATGGGT | | 17 | 26 | 20 | 16 | 29 | 18 | 20 |
| ACCCCG/CTGGGG | | 17 | 20 | 20 | 21 | 23 | 12 | 11 |
| AAAACC/GGTTTT | | 16 | 26 | 19 | 28 | 13 | 14 | 32 |
| AAATTT/AAATTT | | 16 | 19 | 14 | 19 | 13 | 15 | 18 |
| AACACT/ATTGTG | | 16 | 9 | 9 | 18 | 8 | 8 | 15 |
| AACGGG/CCCTTG | | 16 | 28 | 28 | 21 | 21 | 12 | 17 |
| ACACGT/ATGTGC | | 16 | 18 | 17 | 17 | 10 | 13 | 9 |
| ACAGTC/AGTGTC | | 16 | 25 | 20 | 19 | 21 | 18 | 16 |
| ACGATC/AGTGCT | | 16 | 35 | 22 | 16 | 28 | 27 | 29 |
| ACGGCC/CCGGTG | | 16 | 39 | 20 | 25 | 23 | 9 | 8 |
| AGCGGT/ATCGCC | | 16 | 18 | 14 | 7 | 9 | 7 | 12 |
| AAGGGG/CCCCTT | | 15 | 32 | 38 | 30 | 27 | 23 | 23 |
| ACCGGT/ATGGCC | | 15 | 27 | 15 | 23 | 18 | 16 | 10 |
| ACCTAG/ATCTGG | | 15 | 36 | 10 | 29 | 8 | 32 | 6 |
| ACGGGG/CCCCTG | | 15 | 23 | 25 | 19 | 22 | 24 | 19 |
| AACACC/GGTTGT | | 14 | 19 | 12 | 12 | 22 | 14 | 14 |
| AACCTT/AATTGG | | 14 | 6 | 1 | 10 | 12 | 5 | 3 |
| AAGTCC/AGGTTC | | 14 | 10 | 12 | 12 | 7 | 13 | 14 |
| ACATGC/ACGTGT | | 14 | 26 | 13 | 19 | 10 | 17 | 16 |
| ACCGGC/CCGTGG | | 14 | 23 | 14 | 20 | 35 | 16 | 11 |
| ACCTGC/ACGTGG | | 14 | 20 | 18 | 18 | 16 | 16 | 14 |
| ACGAGT/ATGCTC | | 14 | 12 | 19 | 12 | 14 | 13 | 16 |
| AAATAC/ATGTTT | | 13 | 17 | 12 | 9 | 10 | 8 | 2 |
| AACGAT/ATTGCT | | 13 | 25 | 14 | 8 | 17 | 15 | 8 |
| ACGCGC/CGCGTG | | 13 | 16 | 8 | 9 | 16 | 6 | 9 |
| ACTCCC/AGGGTG | | 13 | 15 | 13 | 11 | 11 | 7 | 9 |
| AAATTG/AACTTT | | 12 | 11 | 12 | 20 | 10 | 14 | 13 |
| AACGGC/CCGTTG | | 12 | 20 | 13 | 23 | 16 | 12 | 7 |
| AAGCAC/CGTGTT | | 12 | 13 | 6 | 12 | 14 | 5 | 12 |
| AATGAT/ACTATT | | 12 | 37 | 31 | 35 | 21 | 27 | 28 |
| ACACAT/ATGTGT | | 12 | 25 | 15 | 20 | 22 | 18 | 11 |
| ACCGTC/AGTGGC | | 12 | 17 | 10 | 15 | 16 | 10 | 17 |
| ACGTCG/AGCTGC | | 12 | 20 | 10 | 17 | 17 | 10 | 11 |
| ACTCGT/AGCATG | | 12 | 15 | 12 | 10 | 18 | 16 | 10 |
| AGAGCC/CGGTCT | | 12 | 17 | 16 | 13 | 15 | 7 | 7 |
| AGATCC/AGGTCT | | 12 | 50 | 10 | 37 | 7 | 27 | 9 |
| AACACG/CTTGTG | | 11 | 19 | 14 | 11 | 13 | 7 | 5 |
| AACCAT/ATTGGT | | 11 | 11 | 6 | 8 | 10 | 14 | 10 |
| AACCCC/GGGGTT | | 11 | 22 | 16 | 13 | 12 | 11 | 18 |
| AACTCG/AGCTTG | | 11 | 10 | 12 | 10 | 13 | 11 | 12 |
| AAGCGT/ATTCGC | | 11 | 6 | 6 | 5 | 7 | 9 | 4 |
| ACGACT/ATGCTG | | 11 | 15 | 14 | 8 | 7 | 7 | 5 |
| ACGGGT/ATGCCC | | 11 | 9 | 6 | 8 | 6 | 8 | 6 |
| ACGGTG/ACTGCC | | 11 | 14 | 16 | 19 | 13 | 16 | 26 |
| AGGCTC/AGTCCG | | 11 | 18 | 10 | 17 | 16 | 13 | 8 |
| AACAGG/CCTTGT | | 10 | 12 | 10 | 6 | 12 | 9 | 8 |
| AAGGAC/CCTGTT | | 10 | 20 | 17 | 7 | 12 | 12 | 12 |
| AATACC/ATGGTT | | 10 | 10 | 6 | 4 | 9 | 12 | 5 |
| AATGTG/ACACTT | | 10 | 4 | 8 | 4 | 7 | 4 | 6 |
| ACATCC/AGGTGT | | 10 | 20 | 12 | 13 | 13 | 17 | 18 |
| ACCCTC/AGTGGG | | 10 | 14 | 8 | 6 | 15 | 4 | 1 |
| ACCGCG/CGCTGG | | 10 | 16 | 7 | 15 | 17 | 10 | 4 |
| ACGCCC/CGGGTG | | 10 | 24 | 20 | 14 | 17 | 15 | 21 |
| ACGTAG/ATCTGC | | 10 | 7 | 12 | 14 | 8 | 9 | 8 |
| AGAGCG/CGCTCT | | 10 | 22 | 15 | 15 | 16 | 11 | 20 |
| AAAGAC/CTGTTT | | 9 | 12 | 9 | 10 | 12 | 8 | 14 |
| AAAGTT/AATTTC | | 9 | 14 | 13 | 24 | 9 | 15 | 9 |
| AAGATT/AATTCT | | 9 | 9 | 7 | 3 | 5 | 6 | 8 |
| AATAGC/ATCGTT | | 9 | 13 | 6 | 14 | 10 | 9 | 9 |
| AATCTT/AATTAG | | 9 | 2 | 7 | 3 | 5 | 5 | 6 |
| ACACAG/CTGTGT | | 9 | 10 | 3 | 4 | 8 | 7 | 9 |
| ACGGCT/ATGCCG | | 9 | 14 | 7 | 14 | 12 | 9 | 8 |
| ACGTCT/AGATGC | | 9 | 11 | 15 | 14 | 11 | 8 | 11 |
| AGATCG/AGCTCT | | 9 | 8 | 9 | 11 | 5 | 4 | 9 |
| AGCCCG/CGGGCT | | 9 | 14 | 10 | 20 | 10 | 11 | 3 |
| AAAGTC/AGTTTC | | 8 | 30 | 17 | 12 | 22 | 13 | 18 |
| AACCTG/ACTTGG | | 8 | 19 | 13 | 13 | 17 | 16 | 12 |
| AAGCTC/AGTTCG | | 8 | 7 | 16 | 5 | 18 | 6 | 7 |
| AAGTCG/AGCTTC | | 8 | 8 | 6 | 6 | 8 | 3 | 3 |
| AATATT/AATTAT | | 8 | 16 | 20 | 13 | 7 | 6 | 11 |
| AATCAC/AGTGTT | | 8 | 15 | 10 | 5 | 4 | 10 | 5 |
| AATCTG/ACTTAG | | 8 | 5 | 3 | 2 | 10 | 0 | 7 |
| ACAGGT/ATGTCC | | 8 | 9 | 3 | 5 | 12 | 6 | 6 |
| ACCCTG/ACTGGG | | 8 | 4 | 7 | 4 | 9 | 0 | 4 |
| AGAGCT/ATCTCG | | 8 | 13 | 8 | 9 | 7 | 3 | 9 |
| AGCCCT/ATCGGG | | 8 | 11 | 7 | 3 | 4 | 4 | 1 |
| CCCCCG/CGGGGG | | 8 | 24 | 10 | 30 | 22 | 18 | 4 |
| CCCCGG/CCGGGG | | 8 | 27 | 16 | 17 | 14 | 7 | 7 |
| AAACGT/ATTTGC | | 7 | 6 | 11 | 6 | 9 | 2 | 5 |
| AAAGTG/ACTTTC | | 7 | 9 | 4 | 4 | 17 | 2 | 1 |
| AACATT/AATTGT | | 7 | 10 | 3 | 7 | 7 | 3 | 8 |
| AACCAC/GGTGTT | | 7 | 24 | 14 | 10 | 22 | 14 | 15 |
| AAGCCC/CGGGTT | | 7 | 12 | 10 | 13 | 9 | 7 | 4 |
| AAGGCC/CCGGTT | | 7 | 8 | 3 | 12 | 5 | 4 | 14 |
| AAGGTG/ACTTCC | | 7 | 11 | 18 | 5 | 2 | 6 | 7 |
| AAGGTT/AATTCC | | 7 | 4 | 3 | 5 | 3 | 2 | 4 |
| AATATG/ACTTAT | | 7 | 11 | 2 | 6 | 7 | 5 | 7 |
| AATGCG/ACGCTT | | 7 | 8 | 3 | 5 | 12 | 6 | 5 |
| ACACCT/ATGTGG | | 7 | 26 | 13 | 12 | 17 | 17 | 15 |
| ACAGAT/ATGTCT | | 7 | 19 | 10 | 7 | 6 | 5 | 7 |
| ACATGT/ACATGT | | 7 | 7 | 5 | 2 | 2 | 3 | 1 |
| ACTAGG/ATCCTG | | 7 | 3 | 8 | 1 | 4 | 7 | 9 |
| ACTATG/ACTGAT | | 7 | 10 | 3 | 10 | 5 | 4 | 12 |
| ACTCGG/AGCCTG | | 7 | 20 | 14 | 12 | 12 | 19 | 4 |
| AGGATC/AGTCCT | | 7 | 3 | 9 | 2 | 10 | 6 | 5 |
| AGGGTC/AGTCCC | | 7 | 5 | 1 | 5 | 10 | 1 | 3 |
| AAACAG/CTTTGT | | 6 | 12 | 12 | 10 | 16 | 20 | 13 |
| AACAAT/ATTGTT | | 6 | 7 | 10 | 4 | 6 | 6 | 1 |
| AACGTC/AGTTGC | | 6 | 3 | 4 | 13 | 4 | 6 | 2 |
| AACGTG/ACTTGC | | 6 | 9 | 8 | 11 | 9 | 5 | 7 |
| AACTGC/ACGTTG | | 6 | 11 | 4 | 8 | 1 | 7 | 7 |
| AAGTGG/ACCTTC | | 6 | 10 | 5 | 8 | 3 | 4 | 2 |
| AAGTGT/ACATTC | | 6 | 6 | 5 | 3 | 4 | 2 | 8 |
| AATCGC/AGCGTT | | 6 | 2 | 3 | 5 | 4 | 1 | 4 |
| ACATGG/ACCTGT | | 6 | 10 | 5 | 8 | 5 | 4 | 4 |
| ACCAGT/ATGGTC | | 6 | 3 | 3 | 5 | 6 | 2 | 0 |
| ACCCGT/ATGGGC | | 6 | 10 | 6 | 5 | 9 | 3 | 9 |
| AGCATC/AGTCGT | | 6 | 9 | 13 | 15 | 9 | 7 | 6 |
| AGCGCC/CGCGGT | | 6 | 16 | 4 | 14 | 14 | 8 | 2 |
| AGGCCC/CCGGGT | | 6 | 10 | 3 | 7 | 10 | 0 | 5 |
| AGGGCT/ATCCCG | | 6 | 18 | 4 | 6 | 7 | 6 | 2 |
| AACATC/AGTTGT | | 5 | 16 | 14 | 10 | 12 | 8 | 18 |
| AACCGG/CCTTGG | | 5 | 8 | 10 | 5 | 7 | 8 | 8 |
| AACTAC/ATGTTG | | 5 | 18 | 17 | 18 | 20 | 8 | 12 |
| AAGAGT/ATTCTC | | 5 | 5 | 2 | 4 | 2 | 1 | 0 |
| AAGCTG/ACTTCG | | 5 | 4 | 7 | 5 | 2 | 2 | 3 |
| AAGTCT/AGATTC | | 5 | 7 | 4 | 2 | 3 | 2 | 3 |
| AAGTAT/ATATTC | | 5 | 15 | 7 | 10 | 4 | 6 | 6 |
| AATAGG/ATCCTT | | 5 | 5 | 2 | 3 | 5 | 2 | 2 |
| AATCCT/AGGATT | | 5 | 13 | 4 | 8 | 10 | 5 | 7 |
| ACACCG/CTGTGG | | 5 | 11 | 9 | 6 | 11 | 9 | 6 |
| ACCGAG/CTCTGG | | 5 | 19 | 14 | 11 | 17 | 8 | 16 |
| ACCGGG/CCCTGG | | 5 | 6 | 3 | 5 | 9 | 3 | 1 |
| ACGCGG/CCTGCG | | 5 | 19 | 4 | 9 | 7 | 16 | 4 |
| AGGCGC/CCGCGT | | 5 | 15 | 1 | 4 | 8 | 7 | 5 |
| AGGGAT/ATCCCT | | 5 | 5 | 9 | 7 | 3 | 3 | 2 |
| CCCGCG/CGCGGG | | 5 | 20 | 13 | 13 | 16 | 10 | 8 |
| AAACTC/AGTTTG | | 4 | 4 | 2 | 4 | 3 | 4 | 4 |
| AAATCT/AGATTT | | 4 | 4 | 3 | 3 | 3 | 1 | 0 |
| AACGCT/ATTGCG | | 4 | 5 | 3 | 5 | 1 | 7 | 2 |
| AACTTC/AAGTTG | | 4 | 6 | 11 | 7 | 0 | 2 | 5 |
| AACTTG/AACTTG | | 4 | 1 | 3 | 3 | 4 | 3 | 5 |
| AAGCGG/CCTTCG | | 4 | 13 | 6 | 4 | 16 | 4 | 4 |
| AAGGCG/CCGCTT | | 4 | 16 | 9 | 11 | 12 | 8 | 6 |
| AAGTGC/ACGTTC | | 4 | 6 | 11 | 4 | 2 | 6 | 11 |
| AATCTC/AGAGTT | | 4 | 7 | 4 | 6 | 7 | 5 | 4 |
| ACAGCT/ATGTCG | | 4 | 2 | 2 | 0 | 2 | 6 | 4 |
| ACATAG/ATCTGT | | 4 | 11 | 7 | 3 | 8 | 2 | 3 |
| ACCCAG/CTGGGT | | 4 | 6 | 3 | 6 | 8 | 0 | 3 |
| ACCGCT/ATGGCG | | 4 | 13 | 20 | 6 | 9 | 10 | 7 |
| ACCTGG/ACCTGG | | 4 | 4 | 0 | 7 | 0 | 2 | 2 |
| ACGAGC/CGTGCT | | 4 | 14 | 2 | 9 | 14 | 9 | 5 |
| ACGCGT/ATGCGC | | 4 | 19 | 5 | 13 | 11 | 4 | 12 |
| ACGCTG/ACTGCG | | 4 | 6 | 0 | 5 | 5 | 1 | 0 |
| ACTCGC/AGCGTG | | 4 | 5 | 4 | 2 | 9 | 2 | 9 |
| AGCCAT/ATCGGT | | 4 | 2 | 6 | 1 | 3 | 2 | 2 |
| AGGGCC/CCCGGT | | 4 | 1 | 8 | 8 | 7 | 4 | 5 |
| AGGTCC/AGGTCC | | 4 | 4 | 0 | 3 | 2 | 6 | 6 |
| AGTATC/AGTCAT | | 4 | 12 | 5 | 13 | 8 | 5 | 13 |
| AAGCAT/ATTCGT | | 3 | 4 | 5 | 1 | 3 | 3 | 3 |
| AAGCCG/CGGCTT | | 3 | 12 | 7 | 6 | 3 | 4 | 6 |
| AAGTTC/AAGTTC | | 3 | 3 | 5 | 5 | 1 | 2 | 6 |
| ACACGG/CCTGTG | | 3 | 7 | 2 | 3 | 10 | 2 | 3 |
| ACATCG/AGCTGT | | 3 | 5 | 3 | 3 | 6 | 2 | 1 |
| ACCAGG/CCTGGT | | 3 | 2 | 2 | 2 | 6 | 2 | 2 |
| ACCATG/ACTGGT | | 3 | 1 | 1 | 5 | 2 | 2 | 4 |
| ACCCGG/CCTGGG | | 3 | 13 | 5 | 4 | 6 | 11 | 13 |
| ACCGTG/ACTGGC | | 3 | 8 | 7 | 8 | 3 | 2 | 2 |
| AAACGC/CGTTTG | | 2 | 1 | 2 | 2 | 2 | 0 | 4 |
| AAAGCG/CGCTTT | | 2 | 2 | 4 | 2 | 2 | 2 | 2 |
| AACGTT/AATTGC | | 2 | 7 | 1 | 0 | 2 | 2 | 2 |
| AACTAT/ATATTG | | 2 | 2 | 1 | 6 | 2 | 4 | 1 |
| AACTCT/AGATTG | | 2 | 7 | 6 | 7 | 7 | 8 | 4 |
| AAGGAT/ATTCCT | | 2 | 10 | 3 | 0 | 1 | 2 | 2 |
| AATATC/AGTTAT | | 2 | 3 | 1 | 3 | 4 | 1 | 1 |
| AATCGG/AGCCTT | | 2 | 4 | 2 | 2 | 1 | 3 | 0 |
| AATGCC/ACGGTT | | 2 | 9 | 6 | 4 | 3 | 5 | 4 |
| ACAGCC/CGGTGT | | 2 | 11 | 13 | 11 | 4 | 2 | 4 |
| ACAGGC/CCGTGT | | 2 | 5 | 6 | 7 | 9 | 3 | 3 |
| ACCGAT/ATGGCT | | 2 | 1 | 3 | 5 | 3 | 0 | 3 |
| ACGCAG/CGTCTG | | 2 | 4 | 4 | 3 | 7 | 5 | 4 |
| ACGCCT/ATGCGG | | 2 | 9 | 3 | 6 | 3 | 2 | 1 |
| ACGCTC/AGTGCG | | 2 | 5 | 3 | 6 | 7 | 3 | 4 |
| ACGGTC/AGTGCC | | 2 | 9 | 8 | 5 | 3 | 1 | 1 |
| ACTATC/AGTGAT | | 2 | 2 | 0 | 4 | 5 | 2 | 2 |
| ACTCAT/AGTATG | | 2 | 2 | 0 | 4 | 3 | 4 | 6 |
| AGAGTC/AGTCTC | | 2 | 7 | 4 | 3 | 4 | 4 | 2 |
| AGATCT/AGATCT | | 2 | 4 | 1 | 8 | 2 | 2 | 4 |
| AGCGTC/AGTCGC | | 2 | 3 | 4 | 3 | 4 | 5 | 3 |
| AAATCG/AGCTTT | | 1 | 1 | 5 | 3 | 6 | 3 | 0 |
| AAATGC/ACGTTT | | 1 | 5 | 7 | 4 | 9 | 3 | 9 |
| AAATGT/ACATTT | | 1 | 3 | 3 | 0 | 4 | 4 | 3 |
| AACCCG/CTTGGG | | 1 | 9 | 9 | 11 | 5 | 4 | 5 |
| AACCGT/ATTGGC | | 1 | 7 | 4 | 9 | 4 | 4 | 5 |
| AACGCG/CGCTTG | | 1 | 2 | 0 | 1 | 3 | 2 | 2 |
| AACTGG/ACCTTG | | 1 | 14 | 4 | 2 | 2 | 3 | 2 |
| AACTGT/ACATTG | | 1 | 2 | 0 | 1 | 1 | 0 | 1 |
| AAGCGC/CGCGTT | | 1 | 0 | 2 | 0 | 1 | 2 | 2 |
| AAGGTC/AGTTCC | | 1 | 10 | 3 | 3 | 1 | 0 | 5 |
| AATACG/ATGCTT | | 1 | 7 | 4 | 5 | 1 | 3 | 4 |
| AATCCG/AGGCTT | | 1 | 1 | 3 | 2 | 3 | 1 | 0 |
| AATCGT/AGCATT | | 1 | 2 | 4 | 6 | 5 | 0 | 1 |
| AATGAG/ACTCTT | | 1 | 4 | 2 | 4 | 2 | 2 | 5 |
| AATGCT/ACGATT | | 1 | 0 | 1 | 1 | 1 | 3 | 1 |
| AATGTT/AATTAC | | 1 | 4 | 3 | 3 | 8 | 1 | 4 |
| ACAGGG/CCCTGT | | 1 | 3 | 4 | 10 | 6 | 1 | 1 |
| ACAGTG/ACTGTC | | 1 | 7 | 2 | 2 | 1 | 3 | 2 |
| ACGCAT/ATGCGT | | 1 | 2 | 0 | 4 | 1 | 1 | 1 |
| ACTCAG/AGTCTG | | 1 | 4 | 1 | 0 | 0 | 0 | 2 |
| ACTCTG/ACTGAG | | 1 | 7 | 3 | 4 | 7 | 2 | 3 |
| AGCGCG/CGCGCT | | 1 | 15 | 6 | 10 | 7 | 4 | 3 |
| AGGCAT/ATCCGT | | 1 | 1 | 2 | 0 | 4 | 2 | 1 |
| AGGCCT/ATCCGG | | 1 | 4 | 0 | 1 | 1 | 2 | 0 |
| AGGCGT/ATCCGC | | 1 | 3 | 3 | 2 | 2 | 1 | 0 |
| AAGAC/CTGTT | | 0 | 2 | 5 | 3 | 6 | 3 | 1 |
| AACTC/AGTTG | | 0 | 1 | 1 | 4 | 2 | 0 | 0 |
| ACAGCG/CGCTGT | | 0 | 4 | 5 | 6 | 4 | 4 | 5 |
| AATGTC/ACAGTT | | 0 | 3 | 1 | 1 | 1 | 1 | 0 |
| ACGTGC/ACGTGC | | 0 | 3 | 0 | 1 | 4 | 0 | 4 |
| CCCGGG/CCCGGG | | 0 | 3 | 2 | 0 | 0 | 0 | 1 |
| AAAGCT/ATTTCG | | 0 | 2 | 1 | 6 | 4 | 5 | 2 |
| AAGCTT/AATTCG | | 0 | 2 | 0 | 0 | 1 | 0 | 0 |
| AAGGCT/ATTCCG | | 0 | 2 | 1 | 2 | 2 | 0 | 3 |
| AGCTAT/ATATCG | | 0 | 2 | 5 | 4 | 0 | 1 | 1 |
| ACGGAT/ATGCCT | | 0 | 1 | 3 | 0 | 2 | 1 | 4 |
| AGCGAT/ATCGCT | | 0 | 1 | 0 | 0 | 0 | 0 | 0 |
| AGCGCT/ATCGCG | | 0 | 1 | 1 | 2 | 1 | 2 | 1 |
| AAGCCT/ATTCGG | | 0 | 0 | 2 | 1 | 2 | 1 | 0 |
| ACCC/GGGT | | 0 | 0 | 0 | 0 | 0 | 1 | 0 |
